# Loss of the transcriptional repressor TGIF1 results in enhanced Kras-driven development of pancreatic cancer

**DOI:** 10.1186/s12943-019-1023-1

**Published:** 2019-05-20

**Authors:** Ching-Chieh Weng, Mei-Jen Hsieh, Chia-Chen Wu, Yu-Chun Lin, Yan-Shen Shan, Wen-Chun Hung, Li-Tzong Chen, Kuang-Hung Cheng

**Affiliations:** 1 Institute of Biomedical Sciences, National Sun Yat-Sen University, Kaohsiung, 804 Taiwan; 2 Division of Neurology, Department of Internal Medicine, Kaohsiung Armed Forces General Hospital, Kaohsiung, 802 Taiwan; 3 Department of Surgery, National Cheng Kung University Hospital, Tainan, Taiwan; 4 National Institute of Cancer Research, National Health Research Institutes, Tainan, 704 Taiwan; 5 Department of Medical Laboratory Science and Biotechnology, Kaohsiung Medical University, Kaohsiung, 807 Taiwan

**Keywords:** Pancreatic cancer, TGIF1, Has2, PD-L1, M2 macrophage, Metastatic PDAC model

## Abstract

**Background:**

The TG-interacting factor 1 (TGIF1) gene, which encodes a nuclear transcriptional corepressor of the TGFβ1/Smad signaling pathway, has been implicated in the pathogenesis of various types of human cancer; however, its role in pancreatic ductal adenocarcinoma (PDAC) has yet to be elucidated.

**Methods:**

The expression of TGIF1 in human and murine PDAC specimens were detected by IHC analysis. The functions of TGIF1 in in vivo PDAC growth, dissemination, and metastasis were assessed using conditional inactivation of TGIF1 in well-established autochthonous mouse models of PDAC. Primary cells from TGIF1 null or wild type PDAC mice were examined by assays for cell proliferation, migration, invasion, soft agar and xenograft tumorigenesis. Gene expression profiling, pathway analyses, epigenetic changes associated with TGIF1 loss, and in vitro and in vivo effects of 4-MU were assessed.

**Results:**

Conditional deletion of TGIF1 in the mouse pancreas had no discernible effect on pancreatic development or physiology. Notably, TGIF1 loss induced KrasG12D-driven PDAC models exhibited shorter latency and greater propensity for distant metastases. Deciphering the molecular mechanisms highlighted the TGIF1 loss-induced activation of the hyaluronan synthase 2 (HAS2)-CD44 signaling pathway and upregulation of the immune checkpoint regulator PD-L1 to facilitate the epithelial–mesenchymal transition (EMT) and tumor immune suppression. We also founded that TGIF1 might function as an epigenetic regulator and response for aberrant EMT gene expression during PDAC progression.

**Conclusions:**

Our results imply that targeting the HAS2 pathway in TGIF1 loss of PDAC could be a promising therapeutic strategy for improving the clinical efficacy against PDAC metastasis.

**Electronic supplementary material:**

The online version of this article (10.1186/s12943-019-1023-1) contains supplementary material, which is available to authorized users.

## Background

Pancreatic cancer (PC) is the fourth leading cause of cancer death in the United States, accounting for more than 41,000 deaths per year [1]. Pancreatic ductal adenocarcinoma (PDAC) accounts for approximately > 85% of all PCs, and recent published data have suggested that PDAC might arise from ductal epithelial cells directly or from their common ancestor [2, 3]. Despite the notable progress in understanding the genetic alterations leading to PDAC, very little progress has been made in elucidating the molecular changes involved in the conversion of local benign PDAC to highly metastatic phenotypes [2, 4]. The dissemination of malignant cancer cells from a primary tumor to secondary sites in the body is the major cause of death in patients with cancer. The metastatic spread of tumor cells can occur through various mechanisms, including local tissue invasion, immune suppression, and hematogenous or lymphatic metastasis [5, 6]. Recent data have demonstrated that immune checkpoint signaling plays a decisive role in mediating tumor promotion in the immune suppressive microenvironment in many solid tumors [7, 8]. Upregulation of immune checkpoint molecules is a strategy that tumors use to escape attack by host immune cells [9]. Programmed cell death receptor ligand 1 (PD-L1), a ligand for checkpoint receptor PD-1 on T cells, is often highly expressed on many tumor cells [10]. Activation of the PD-1 pathway on T cells through high PD-L1 expression on cancer cells attenuates T-cell activation signaling and inhibits host antitumor response [11]. Moreover, PD-1- and PD-L1-blocking antibodies have been demonstrated to be clinically effective in treating many cancers; however, the detailed regulation mechanisms of PD-L1 expression on cancer cells is not well characterized [12, 13].

TGFβ suppresses the cell cycle progression of epithelial cells and inhibits tumor growth; however, in later stages of tumor development, tumor cells generally develop resistance to TGFβ1-mediated growth inhibition, and TGFβ ceases to function in tumor suppression and consequently adopts the converse role of inducing distal metastatic spread [14, 15]. In the canonical TGFβ1 pathway, homodimers of TGFβ1 ligands bind to the TGF-β type 2 (TβR2) receptor, which interact with the TβR1 receptor and phosphorylate it to form a tetramer ligand–receptor complex and then recruit receptor-specific R-Smads (Smad2/3) to be phosphorylated by the receptor complex. The phosphorylated R-Smads are further released from the receptor complex to bind to the comediator Smad, Smad4, and translocate to the nucleus to activate the TGFβ1 response genes [16]. Smad-dependent signaling induced by TGF-β1 is tightly modulated by negative regulatory mechanisms through inhibitory Smads (I-Smads) (Smad6 and − 7) and through the recruitment of transcriptional corepressor complexes such as c-Ski, SnoN, and TG-interacting factor 1 (TGIF1) [17, 18]. Thus, TGIF1 has been identified as a member of the three-amino acid loop extension class of homeodomain proteins that function as corepressors of the TGF-β1 pathway [17, 19]. TGIF1 is normally located in the nucleus; it can be induced by TGFβ/Smad signaling and acts as the negative corepressor by interacting with Smad2, Ski/Sno repressors, and HDAC to form an inhibition complex of TGF-β-activated Smad transcriptional machinery [18, 20]. In general, TGIF1 regulates TGFβ1 signaling through at least two mechanisms: 1) increase in I-Smad expression, leading to an increase in Smad7 expression, which interferes with the TGFβ receptor by blocking the interactions between the R-Smads and the activated receptor complex [21]; 2) suppression of R-Smad gene expression by inhibitory cofactors Sno, Ski, and TGIF1 [18, 22]. In addition, a recent study suggested that TGIF1 functions as a component of a ubiquitin ligase complex to mediate the degradation of Smad2 in response to TGF-β signaling [21, 22]. Based on these reports, we hypothesized that TGIF1 deficiency or downregulation is an alternative mechanism underlying the late-stage TGFβ1/Smad-mediated tumor progression and metastasis.

Recent research with genetically engineered mouse (GEM) models has identified the combinatorial roles of tumor-associated gene inactivation/mutations in tumor development. Here, we extend our previous GEM study on PDAC to incorporate TGIF1 conditional floxp mice into the Pdx-1Cre-driven Kras^G12D^ PDAC model and to determine the effect of conditional TGIF1 deletion on PDAC development. We crossed the conditional TGIF1 floxp allele mice with the Pdx-1Cre Kras^G12D^ and Pdx-1Cre Kras^G12D^p53 ^loxp/loxp^ compound mice to determine the effect of TGIF1 loss on the overall PDAC development and metastatic behaviors [2, 23]. We found that loss of TGIF1-null PDAC resulted in a high EMT and suppression of antitumor immunity phenotypes. Furthermore, our data revealed the role of TGIF1 in regulating TGFβ-induced hyaluronan synthase 2 (HAS2) activity, which has a profound effect on tumor-associated macrophage (TAM) polarization; moreover, TGIF1 exhibits an antitumor immunity potential through the suppression of PD-L1 expression. In summary, this in vivo study provides insights into the role of TGIF1 of the TGFβ/Smad signaling axis in PDAC development and its therapeutic potential.

## Methods

### Genetically modified mice and mouse genotyping

Pdx-1Cre, LSL-Kras^G12D^ and p53^Loxp/Loxp^ mice, obtained from the Mouse Models of Human Cancers Consortium (MMHCC) under material transfer agreements, were generously made available by Drs Andrew M Lowy, Tyler Jacks and Anton Berns respectively [24]. TGIF1^Loxp/Loxp^ mice were purchased from the Jackson Laboratory under material transfer agreements, were generously made available by Christopher Walsh [19]. Mutant mice were genotyped as described by the MMHCC and Jackson Laboratory PCR protocols for strains 01XL5, 01XJ6, 01XC2 and #021443. Mice were maintained on a mixed genetic background of 129/Sv and FVB/N. All studies were approved by the Animal Care Committee of the National Sun Yat-Sen University (animal permit number 10440) and all surgery and killing was performed using isoflurane or avertin to ensure minimal suffering. Pancreatic tissue samples were fixed in 10% buffered formalin overnight, washed with 1 × phosphate-buffered saline, and transferred to 70% ethanol before paraffin embedding, sectioning, and hematoxylin and eosin staining.

### Immunohistochemistry (IHC) and immunofluorescence (IF)

Hematoxylin and eosin staining followed the standard protocol. Alcian blue staining and Masson’s trichrome staining kits were purchased from Scy-Tek Laboratories (Logan, UT, USA) and performed according to the manufacturer’s protocols. Standard procedure for IHC and immunofluorescence analysis has been described in detail previously and antibodies used in these studies are listed in Additional file 1: Table S1 [25]. Stained slides were captured using a Carl Zeiss. Axioskop 2 plus microscope (Carl Zeiss, Thornwood, NY, USA). Immunofluorescence (IF) images were captured using a Delta Vision Personal DV Imaging System (Personal DV Applied Precision, Issaquah, WA, USA).

### Human tissue array analysis

For cytokine analysis, the Pancreatic cancer tissue array (Cat: PA807) was purchased from US Biomax Inc., Rockville, MD, USA. The tissue sections were immune-stained in a regular way using anti-TGIF1 antibody. H&E and IHC images were evaluated from the Department of Pathology in the National Cheng Kung University Hospital using an IRB approved protocol (IRB:B-ER-104-011).

### Western blot analysis

Standard procedures for immunoblotting analysis has been described in detail previously. The primary antibodies used in this study are listed in Additional file 1: Table S1 [25].

### Mouse cytokine array analysis

For cytokine analysis, the RayBio Mouse Cytokine Array C3 (Cat: AAM-CYT-3-4) was purchased from RayBiotech, Inc., Norcross, GA, USA. Sample preparation and hybridization to the array were performed according to the manufacturer’s instructions.

### Real-time–quantitative PCR analysis (RT–qPCR)

RT–qPCR was carried out as described in detail previously, and the primers for RT–qPCR were listed in Additional file 1: Table S2.

### Cell proliferation assay

Standard methyl tetrazolium-(MTT) based cell growth assay as described in detail previously [25].

### Wound-healing assay

Cells were pretreated with 0.02% (0.2 mg/mL) mitomycin C for 2 h and wounded by removing a 300–500 mm wide strip of cells across the well with a standard 200 mL yellow tip. Wounded monolayers were washed twice with phosphate-buffered saline buffer solution to remove non-adherent cells. The cells were cultured in low FBS media and incubated for predetermined times to monitor wound closing. Wound closure was recorded by phase-contrast microscopy according to previously published protocols [25].

### Glucose tolerance test (GTT)

Animals were fasted overnight, and their fasting level of blood glucose was evaluated with an ACCU-CHEK Active (Roche). Mice were then injected IP with 1.5 mg/g of body weight of glucose, and blood glucose were measured at the indicated times.

### Flow cytometry

The protocol for FACS analysis as described in detail previously. All flow cytometry analyses were performed with a BD Accuri C6 Flow Cytometer (BD Biosciences, San Jose, CA) according to the manufacturer’s instructions.

### Complementary DNA microarray analysis

Hybridization was performed against the Affymetrix GeneChip MoGene 1.0 ST array. The arrays were hybridized for 17 h at 45 °C and 60 r.p.m. Arrays were subsequently washed (Affymetrix Fluidics Station 450, Santa Clara, CA, USA) and stained with streptavidin–phycoerythrin (GeneChip Hybridization, Wash, and Stain Kit, Affymetrix, Santa Clara, CA, USA; 900,720), and scanned on an Affymetrix GeneChip Scanner 3000. The resulting data were analyzed using Expression Console software (Affymetrix) and Transcriptome Analysis Console software (Affymetrix) with default RMA parameters. Genes regulated were determined with a 2.0-fold change; *P*-value< 0.05.

### GeneGo analysis

The significant lists were uploaded from a Microsoft Excel spreadsheet onto Metacore 6.13 software (GeneGo pathways analysis; https://clarivate.com/products/metacore/). GeneGo recognizes the Affymetrix identifiers and maps the tissues to the MetaCore data analysis suite, generating maps to describe common pathways or molecular connections between pancreatic tissues on the list. Graphical representations of the molecular relationships between genes were generated using the GeneGo pathway analysis, based upon processes showing significant (*P* < 0.05) association.

### Murine primary PDAC cell culture, cytokine and inhibitor treatment

The mouse primary pancreatic cancer cells were cultured in RPMI-1640 medium supplemented with 10% fetal bovine serum, nonessential amino acids, 100 units/ml penicillin and 100 μg/ml streptomycin at 37 °C in a 5% CO2 incubator. Primary mouse prostate glandular and PDAC cells were maintained for < 6 passages and histopathologically characterized through SCID mice xenograft studies before performing microarray expression profile analyses. Spheroids were created using Perfecta3D® Hanging Drop Plates (Sigma Aldrich, St. Louis, MO, USA). Spheroids of cells (2 × 10^3^ cells) were prepared as described above. Cells were treated with the 4-Methylumbelliferone (4-MU; M1381) was obtained from Sigma Aldrich (St. Louis, MO, USA). TGF-β (240-B) was purchased from Selleck Chemicals.

### Xenograft severe combined immunodeficiency (SCID) and allogeneic mice

Specific pathogen-free, 8-week-old female C.B17/lcr-SCID mice were purchased from BioLASCO Taiwan Co., Ltd., for the in vivo tumorigenicity study. For subcutaneous (SQ) tumor allogenic mouse models, 8 week old, male C57BL/6 (B6) mice, mice (BioLASCO Taiwan Co., Ltd) were injected SQ with 5 × 10^5^ PDAC cells (PKP and PKTP) in 100ul sterile PBS. The animals were maintained in the animal center at the Department of Biological Science, National Sun Yat-Sen University under SPF conditions and treated according to the institutional guidelines for the care and use of experimental animals (animal permit number 10639).

### In vivo 4-Methylumbelliferone (4-MU) treatment

For in vivo treatment, 4-Methylumbelliferone (4-MU) (30 mg) was dissolved in 1 ml of dimethyl sulfoxide (DMSO) solution to a final concentration of 30 mg/ml directly before use. Six-week-old PKP(P53^Loxp^/^+^) and PKTP(P53^Loxp^/^+^) mice were administered 450 mg/kg 4-MU twice weekly via intraperitoneal injections for 3 weeks and compared to DMSO treated vehicle control mice (*N* > 3 per group). At the end of the experiment, mice were sacrificed by anesthetizing with avertin, and their pancreata and intraabdominal organs were collected for pathohistological analysis. Randomization was done according to genotype and blinding was applied during histological analysis [26].

### Human PDAC specimens

Primary PDAC tissues were collected from participants who consented and enrolled with institutional review board approval from the National Cheng Kung University Hospital, Tainan, Taiwan (IRB:B-ER-104-011). Primary tumors were snap frozen immediately after resection until further analysis.

### Genomic DNA isolation and bisulfite treatment

Genomic DNA from primary PDAC tissues and cell lines were isolated using a DNeasy Tissue Kit (QIAGEN,Hilden, Germany) according to the manufacturer’s instructions. Standard procedures for Genomic DNA isolation has been described in detail previously [27]. Commercial human genomic DNA from normal pancreas and pancreatic ductal adenocarcinoma were purchased from Origene (CD564366, CD565370, CD564282, CD563905, CD563941, and CD564012; Rockville, MD).

### Methylation-specific polymerase chain reaction (MSP)

The TGIF1 promoter has been described in one previous study using the upstream promoter area for the amplification of defined CpG islands containing promoter DNA sequences of either unmethylated (UMSP) or methylated (MSP) DNA sequences after sodium bisulfite treatment. Sequences of the forward and reverse MSP and UMSP primers used in this study were listed in Additional file 1: Table S3. Polymerase chain reaction (PCR) conditions were 95 °C for 5 min, 35 cycles of 95 °C for 30 s, 58 °C for 30 s and 72 °C for 30 s, followed by a final extension at 72 °C for 5 min. A 10-μL sample of each PCR product was mixed with 1 × loading buffer and analyzed by electrophoresis on non-denaturing 8% polyacrylamide gel and visualized by staining with ethidium bromide [27].

### Lentivirus production and shRNA for gene knockdown

The plasmids required for shRNA lentivirus production were purchased from the National RNAi Core Facility, Academia Sinica, Taiwan. The pLKO.1-shRNA vectors used for knockdown of TGIF1, ETV1 and HAS2 were TRCN0000020149 (TGIF1), TRCN0000337811 (HAS2) and TRCN0000075475 (ETV1). The pLKO.1-shEGFP control plasmid was TRCN00000–72190 (EGFP). Lentivirus production and infection were performed according to a previously described protocol [25].

### Chromatin immunoprecipitation (ChIP) assay

ChIP assays with the anti-H3K27 acetylation antibody on PKP and PKTP cells in the absence or in the presence of SAHA (10 μM) were performed using the High-Sensitivity ChIP Kit (ab185913; Abcam, Cambridge, UK) according to the manufacturer’s instruction. Precipitated DNA was analyzed using Real Time- qPCR primers were listed in Additional file 1: Table S2.

### Statistical analysis

All experiments were repeated at least three times. One representative experiment is shown. RT–qPCR and cell proliferation assays are displayed as one representative experiment of three independent experiments, mean ± s.e.m. Data measured on continuous scale were analyzed using Student’s t-test and categorical data were subjected to × 2 test. *P*-value < 0.05 was considered significant.

## Results

### Pancreas-specific TGIF1 deficiency in mice does not alter pancreatic development or induce tumorigenesis

**Fig. 1:**
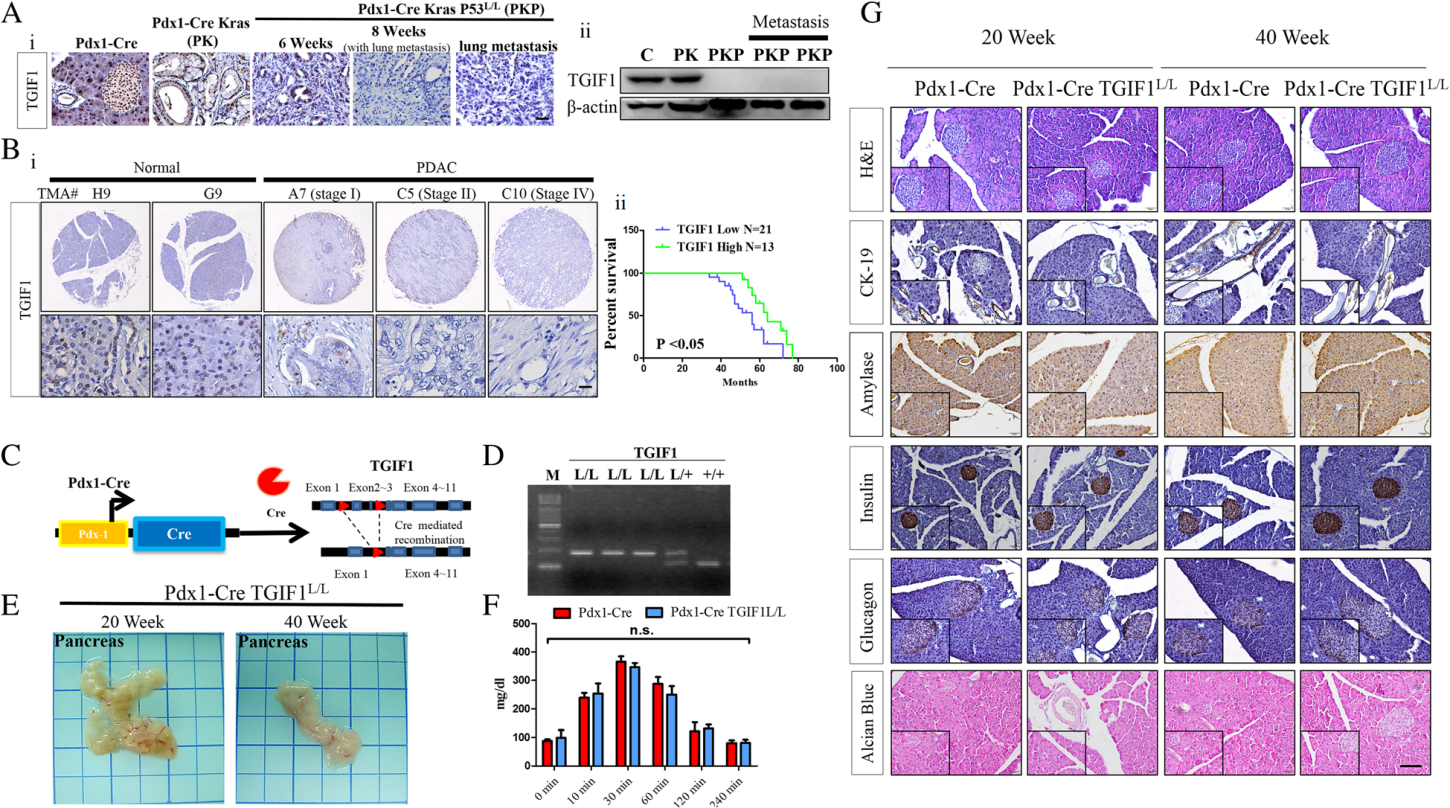
TGIF1 expression in PDAC and TGIF1 ablation in the pancreas do not interrupt pancreatic development in mice. **a** IHC (i) and western blot (ii) analyses of TGIF1 protein expression in mouse normal pancreata, PanINs, and PDAC tissues from mice with indicated genotypes. **b i.** TGIF1 expression in the human PDAC tissue microarrays (TMAs), as shown by IHC analysis. **ii***.* Kaplan-Meier survival curves of low and high expression TGIF1 groups. *P < 0.05. **c** Schematic indicating the targeted TGIF1 allele before and after Cre-mediated recombination. **d** PCR genotyping reactions detected the wild-type and TGIF1 loxp alleles from the tail DNA of mice littermates. **e** Gross anatomy of the pancreas in wild-type and Pdx-1CreTGIF1^L/L^ mice. **f** Glucose tolerance test (GTT) determined the blood glucose concentration in wild type and Pdx-1CreTGIF1^L/L^ mice. n.s stands for not statistically significant. **g** Representative images of hematoxylin and eosin (H&E) staining and immunostaining detection of the epithelial marker cytokeratin19 (CK19), islet enzymes insulin and glucagon, acinar marker amylase and Alcian blue stain in the pancreata of wild-type and Pdx-1Cre TGIF1^L/L^ mice (insert 400 x). Scale bar = 100 μm

In the normal pancreas of mice, TGIF1 was observed in the nuclei of all pancreatic lineage cells. Moderate to high nuclear TGIF1 expression levels were detected in the pancreatic intraepithelial neoplasias (PanINs) of Pdx-1Cre Kras^G12D^ mice; however, a markedly reduced TGIF1 staining intensity was observed in the primary and metastatic PDAC of Pdx-1CreLSL-Kras^G12D^p53^L/L^ (PKP) mice (Fig. 1Ai-ii). Similar results were obtained from IHC staining for TGIF1 expression on human PDAC tissue microarrays, implying that TGIF1 is usually inactivated at later stages of human PDAC (≧stage II), and reduced TGIF1 expression exhibited reduced overall survival compared with patients whose PDAC had higher levels of TGIF1. (Fig. 1Bi-ii, Additional file 2: Figure S1). Thus, the significantly reduced TGIF1 protein expression in PDAC led us to conduct additional experiments to assess the biological function of TGIF1 in PDAC development.

To investigate the relevance of TGIF1 ablation in pancreatic development and homeostasis, we obtained conditional TGIF1 knockout mice from the Jackson Laboratory (Maine, USA), and crossed them with the Pdx-1Cre transgenic strain. The Pdx-1Cre transgenic mice were established by Dr. Douglas Melton’s laboratory to specifically express Cre recombinase in the endodermal pancreatic progenitors in postnatal fetal mice (E9.5-E11) (Fig. 1c) [28]. Polymerase chain reaction (PCR) genotyping analysis was performed to confirm the TGIF1 floxp band and Cre transgenic alleles in the littermates (Fig. 1d). We also confirmed the deletion of TGIF1 expression through Western blot and IHC analyses (data not shown). Pdx-1CreTGIF1^L/L^ mice were born in accordance with the Mendelian ratios and grew to > 40 weeks with no visible phenotypic effects (Fig. 1e). Pancreatic lineage-specific TGIF1 deficiency appeared grossly normal and did not cause any pancreatic morphological defect, as demonstrated histologically (Fig. 1e, g). Furthermore, a glucose tolerance test (GTT) revealed no differences in blood glucose levels between wild type and these compound mice (Fig. 1f). IHC analysis also showed that the expression levels of endocrine and exocrine markers were normal in the pancreas of the Pdx-1Cre TGIF1^L/L^ mice (Fig. 1g). Based on the observations of the histological and glucose tolerance examinations in the pancreas of the Pdx-1CreTGIF1^L/L^ mice (*N* = 16), we proposed that pancreas-specific TGIF1 deficiency does not lead to any pancreas developmental defect or result in neoplasms.

### Activated KrasG12D mutation coupled with TGIF1 loss is sufficient for initiating PDAC in mice

**Fig. 2:**
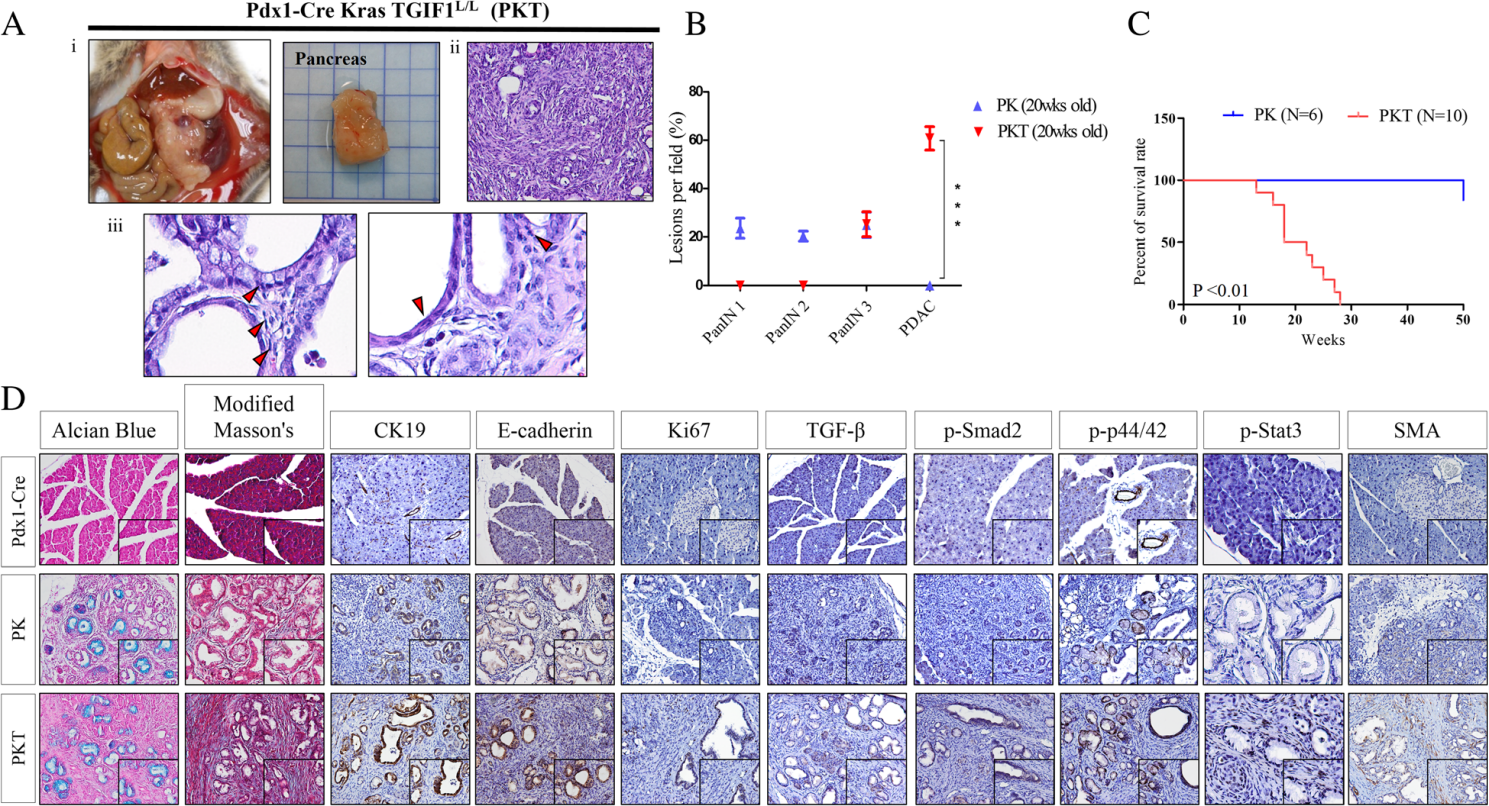
Conditional TGIF1 deletion accelerates progression to PDAC in cooperation with Kras^G12D^. **a i,** Gross morphology of PDAC in 20-week-old Pdx-1Cre Kras^G12D^TGIF1^L/L^ (PKT) mice. **ii,** Representative H&E staining images of tissue paraffin sections showing well-differentiated PDAC in PKT mice. **Iii,** High-power images revealing abnormal anaplastic nuclear features in abundance. **b** Quantitation of histopathological alterations in the indicated phenotypes of pathology ***p < 0.001. **c** Kaplan–Meier curves showing the percentage of survival rates of the indicated genotypes (*P* < 0.01). **d** PDAC tissue sections of the indicated genotype were stained with Alcian blue and elastic tissue-Masson stain; IHC analysis revealed staining for the proliferating marker Ki67; epithelial markers, CK19 and E-cadherin (E-cad), TGFβ1/p-Smad2, kinase pathway proteins, p-Akt, p-STAT3 and pErk (p-p44/42)and stromal markers, SMA (insert 400 x). Scale bar, 100 μm

According to our IHC data, TGIF1 expression was initial upregulated in the PanINs; however, reduced expression or expression inactivation was observed in advanced PDAC or metastatic PDA, compared with nontumorous pancreatic tissues. Additionally, TGF-β signaling is considered to have highly context-dependent effects on PanINs and PDAC formation [29]. To further exploit the requirement of TGIF1 in Kras-driven PainIN in the vivo mouse model, we crossed our conditional TGIF1 mice with Pdx-1CreKras^G12D^ (PK) mutant mice. We examined a cohort of Pdx-1CreKras^G12D^TGIF1^L/L^ (PKT) mice older than 18–20 weeks (Fig. 2a). In contrast to PK mice, which exhibited small and focal PanIN lesions by 18 weeks of age, we observed that all PKT mice developed abdominal distension and succumbed to tumor burden by 18–20 weeks, compared with the 100% PDAC-free condition of the PK model (Fig. 2 b, c). We further compared the pancreatic sections between PKT and PK mice to examine the presence of different grades of PanINs and PDAC lesions (Fig. 2Aii-iii). Histological analysis confirmed the PDAC formation in PKT mice, which was characterized by almost complete loss of normal pancreatic architecture and well-differentiated ductal tumors histologically similar to the human disease (Fig. 2 Aii, b). The specimens of the pancreatic lesions were first subjected to Alcian blue and Masson’s trichrome staining to mark mucin-containing PanIN and PDAC lesions (Fig. 2d). IHC analysis demonstrated that the pancreatic malignant lesions in PKT mice were positive for the epithelial markers cytokeration-19 (CK19) and E-cadherin, and, in particular, tumor cells increased staining for the cell proliferation marker Ki67 were higher in PKT lesions than in the age-matched PK mice (Fig. 2d). Furthermore, the increased IHC staining for TGβ1/p-Smad2, pAkt, p-p44/42, p-Stat3 and SMA were observed in the PDAC of PKT pancreata, compared with those of the PK pancreatic lesions (Fig. 2d). However, an autopsy analysis of the PKT mice at the age of 18–20 weeks revealed that none of these mice developed micrometastasis, which may be attributed to our early termination approaches. In summary, we demonstrated that TGIF1 deficiency in the pancreas is sufficient to induce PDAC in cooperation with KrasG12D.

### TGIF1 loss accelerates PDAC development with reduced survival and increased metastatic behaviors

**Fig. 3:**
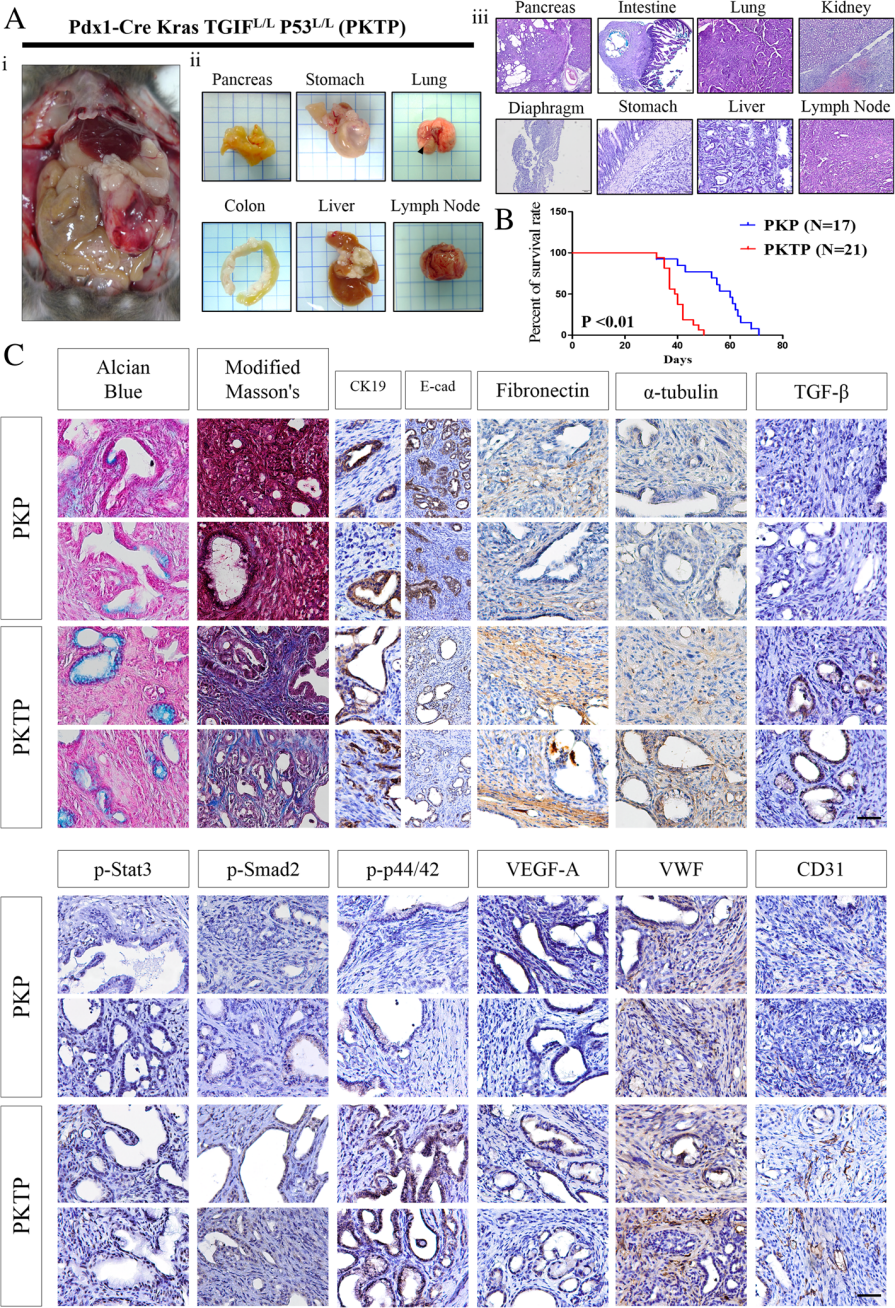
Conditional TGIF1 deletion promotes the development of highly metastatic PDAC in Kras^G12D^ P53^L/L^ models. **a** i, The autopsy image of a 7-week-old PKTP mouse. **ii**, Gross images of multi- organ metastasis in the PKTP mcie (arrows: tumor nodule). **iii,** Histological H&E staining analysis of PDAC and metastatic nodules in secondary disseminating organs of the PKTP mice reveals focal neoplasm lesions with PDAC histology. **b** Kaplan–Meier survival curves of the PKP and PKTP mice (P < 0.01). **c** Alcian blue, modified Mason’s stain and IHC staining for CK19, fibronectin, α-tubulin, E-cad, TGFβ1, p-Smad2, p-STAT3, pAkt, p44/42, VEGFA, VWF and CD31 in PDAC from the PKP and PKTP mice. Scale bar, 100 μm

We and other researchers have established a clear invasive PDAC model with Kas^G12D^ and P53 floxp/floxp alleles [30]. To further determine the consequences of TGIF1 deletion in the regulation of PDAC metastasis, compound Pdx-1CreKras^G12D^ TGIF1^L/L^p53^L/L^ (PKTP) mice were produced and the metastatic behaviors were evaluated in comparison with Pdx-1CreKras^G12D^p53^L/L^ (PKP) mice. Notably, the PKTP (*N* = 21) sibling cohort of mice developed metastatic PDAC much faster than PKP mice, and the survival curve for the cohort of PKTP mice was significantly lower than that of the PKP mice (Fig. 3 a and b). Generally, most PKTP mice developed ascites (*P* < 0.001) and PDAC metastasize to lung (*P* = 0.0071), liver (*P* = 0.006) and lymph node (*P* = 0.01) are seen more frequently than PKP mice (Additional file 1: Table S4). Histological and histochemical (Alican blue and modified Masson’s staining) analyses of these tumors in moribund compound mice demonstrated the epithelial differentiation type, and IHC staining for CK19 was positive in the tumor cells (Fig. 3c). The murine PDAC lesions in the PKTP mice showed low epithelial marker E-cadherin (E-cad) protein expression, but strong immunoreactivity for active stromal response and were positive for fibronectin, α-tubulin and vimentin expression, in contrast to the PDAC derived from the PKP model (Fig. 3c and data not shown). Moreover, evident increases in active TGFβ1/p-Smad2, p-Erk (p-p-44/42) and p-Stat3 were also observed through IHC analysis in the PDAC tissues derived from the PKTP model, compared with those of the PKP mice (Fig. 3d). Furthermore, high expression levels of VWF, VEGFA and CD31 were observed in the PDAC of the PKTP mice through IHC analysis (Fig. 3d). Intriguingly, we also observed that the PKTP mice developed duodenal polyposis similar to that previously we reported in Pdx-1CreKras^G12D^ SMAD4^L/L^ model. [29]

### TGIF1 loss alters tumor microenvironment to suppress tumor immune response in PDAC

**Fig. 4:**
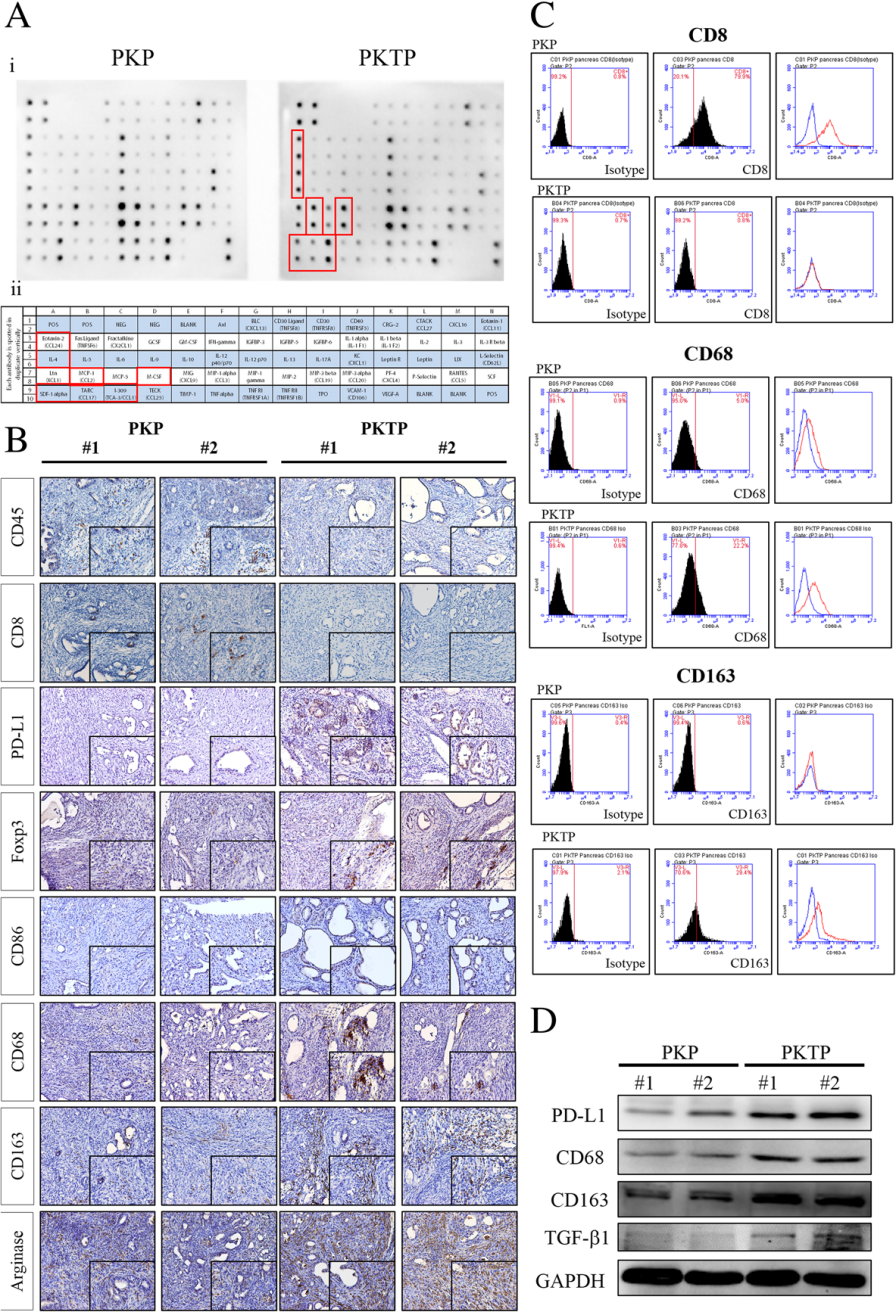
Modulation of the inflammatory cytokine profile and tumor immune response in the PDAC microenvironment by TGIF1. **a i,** Mouse cytokine arrays profile the cytokine expression levels in PDAC of the PKP and PKTP mice. **ii**, Template alignment of the mouse cytokines in the array represent: POS, positive; NEG, negative; IL, interleukin; C-C motif chemokine ligand (CCL); SDF-1, stromal cell-derived factor 1; BLC, B-lymphocyte chemo- attractant; TAC, protachykinin; TCA-3, small inducible cytokine A1; TIMP, tissue inhibitors of metalloproteinase; LIX, LPS induced CXC chemokine; MCSF, macrophage colony stimulating factor; MCP-1, monocyte chemotactic protein 1; MIG, mitogen-inducible gene; MIP-1, macrophage inflammatory protein 1. **b** IHC staining of pancreata from 7-week-old PKP and PPKTP mice with antibodies to detect CD45, CD8, PD-L1, Foxp3 and CD86 (macrophages), CD68, CD163 and arginase (insert 400 x)**.** Scale bar, 100 μm. **c** Detection of the T-lymphatic marker CD8 and M2-macrophage marker CD68; CD163 expression in the PKP and PKTP PDAC tissues as determined by flow cytometry. The data represent three different experiments. **d** Western blot analysis for tumor lysate demonstrated increased expression of PD-L1, CD68, CD163 and TGFβ1 in PDAC of the PKTP mice, compared with that in PDAC of PKP mice

Notably, the PDAC sections derived from PKTP exhibited a strong response of desmoplasia, commonly shown in human PC, compared with the PKP model. Moreover, tumorigenesis-modulated immunogenicity may accompany tumor development under the tissue-specific and oncogene/tumor suppressor-driven manner to reprogram the tumor microenvironment. We observed intensive stromal reactions, which may involve numerous fibroblasts, leukocyte infiltration, and collagen and mucin production in the PDAC lesions of the PKTP mice as compared with those in the PKP model (Fig. 3c); therefore, we tend to screen the elevated levels of cytokines in the PDAC in response to TGIF1 loss. We then employed the mouse cytokine protein array to detect different chemokine receptors or cytokines protein expression levels between the PDAC tissue lysates from the PKP and PKTP models (Fig. 4a). The expression levels of IL-4, M-CSF, CCL2, CCL24, SDF-1 and CCL17 substantially increased in the TGIF1-loss murine PDAC, whereas the expression levels of IL-1, IL-12, TNF, and LIX decreased in PKTP PDAC, compared with those in the PKP model (Fig. 4a).

Next, we investigated the effects of TGIF1 deficiency on leukocyte infiltrations in the tumor microenvironment; IHC and flow cytometry (FACS) analyses were employed to determine the numbers of leukocytes in the stromal tumor microenvironment of the PKTP mice compared with that of the PKP model. We observed a prominent reduction in CD45+ cell infiltrations in the pancreata of the PKTP mice, compared with the PDAC of the PKP mice, as revealed by IHC analysis (Fig. 4b). Similarly, we detected reduced levels of CD8+ cells in the tumor microenvironment of PDAC derived from the PKTP model, whereas a higher staining intensity of CD8+ cells was observed in the pancreata of the PKP mice (Fig. 4b). Because of the reduced T-cell population in the microenvironment of PDAC in the PKTP mice, we further performed IHC using the anti-PD-L1 αAb to detect PD-L1 expression in our PDAC models. Notably, we observed a statistically significant increase in PD-L1 staining activity in the stromal reactions of the PKTP mice, compared with that of the PKP model (Fig. 4b). Our data also revealed that the upregulation of the regulatory T-cells (Tregs) associated marker, Foxp3, at the tumor stromal interface of PDAC derived from the PKTP mice, compared with that of the PKP model.

Accordingly, the similar levels of M1 macrophage marker CD86 were observed in the tumor microenvironment of PKTP and PKP PDAC. Notably, our data revealed increased M2 macrophage markers, CD68 and CD163 and Arginase (Arg), immunoreactivity at the tumor stromal interface of PKTP PDAC, compared with the PKP model (Fig. 4b). Similar results were obtained using FACS analysis and Western blot analysis with murine PDAC tissues, indicating a reduced CD8 staining activity, but increased PDL-1, CD68, CD163, matrix metalloproteinase (MMP) and TGFβ1 expression in PKTP PDAC when compared with that in PKP PDAC (Fig. 4 c, d and data not shown). Collectively, our results imply that TGIF1 may play a role in the regulation of PD-1/PD-L1 signaling axis of the tumor microenvironment, thereby influencing pancreatic tumorigenesis.

### TGIF1-deficient PDAC exhibits enhanced EMT program and cancer stem cell-like phenotype

**Fig. 5:**
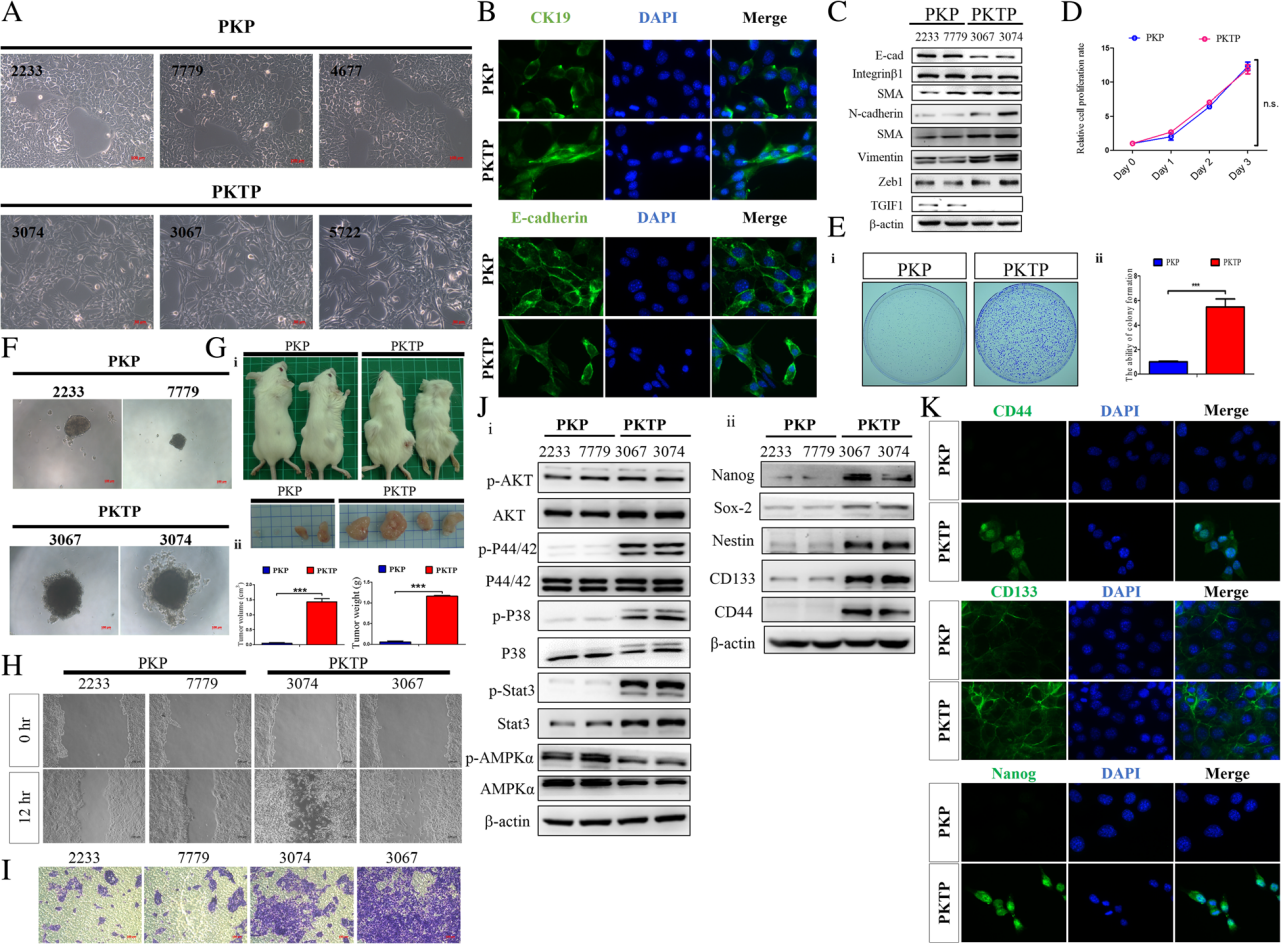
TGIF1 loss promotes EMT and CSC activity to increase PDAC cell migration and invasiveness. **a** Morphological characterization of primary PDAC cells derived from PKP and PKTP mice through phase-contrast microscopy. Scale bar =100 μm. **b** Immunocytochemical analysis of epithelial markers, CK19 or E-cadherin, in PKP and PKTP PDAC cells. Primary cell cultures of PDAC cells were stained for CK19 and E-cadherin. Scale bar =100 μm. **c** Western blot analysis by using the total cell lysate of murine PDAC cells derived from PKP and PKTP mice revealed a decrease in E-cadherin, integrin β1 expression, TGIF1 expression, and an increase in SMA, N-cadherin, Vimentin, and Zeb1 expression in PKTP cells as compared to PKP cells. **d** TGIF1 loss has no significant effect on PDAC cell proliferation in vitro. Cell proliferation was determined using the MTT assay. Data are represented as mean ± s.d; *N* = 3 independent experiments. **e**&**f** TGIF1 significantly inhibits the in vitro tumor sphere-forming ability of PDAC cells, whereas PKTP cells enhanced the anchorage-independent growth **e** and tumor sphere formation in “sphere”-forming assays **f**, compared with PKP cells. **g i,** Engrafted PDAC formation of PKTP and PKP PDAC cells in a SCID mouse xenograft model. **ii**, Average tumor weights and volumes/sizes of the PKTP groups significantly increased compared with the control PKP groups ***p < 0.001.. **h**, Wound healing assays showed that TGIF1 loss stimulates in vitro cell motility of murine PDAC cells. **i** Transwell invasion assays demonstrated that TGIF1 gene ablation enhances the invasive ability of murine PDAC cells. Representative images are from three independent experiments. **j** Western blot analysis of the total and phosphorylated Akt, Erk (p-44/42), STAT3, p38 MAP kinase pathways and cancer stemness marker Nanog, Sox2, Nestin, CD44, and CD133 in PKP and PKTP PDAC cell lines. β-actin served as a loading control. **k** Detection of the stem-cell-specific markers CD44, CD133 and Nanog in PKP and PKTP PDAC cells as determined using immunocytochemical analysis. Scale bar = 100 μm

To more thoroughly characterize the molecular mechanisms underlying TGIF1 inactivation-induced PDAC metastasis, we further isolated primary PDAC cells from the PKP and PKTP models (Fig. 5a). The observed morphological characteristics of the epithelial primary PDAC cells were confirmed through western blotting and immunostaining (Fig. 5 b, c). TGIF1 loss in PDAC cells suppressed the protein expression levels of E-cadherin and Integrin β1, and increased the levels of N-cadherin, SMA, Vimentin and Zeb1 protein expression (Fig. 5c). Next, we determined whether TGIF1 loss modulates PDAC cell proliferation in vitro by using the MTT cell proliferation assay. In contrast to our expectations, TGIF1 deficiency did not significantly influence the murine PDAC cell growth rate in vitro (Fig. 5d) However, TGIF1 deficiency leads to murine PDAC cells displayed resistance to TGF-β-mediated growth inhibition (Additional file 2: Figure S2A). Moreover, the effect of TGIF1 inactivation on the tumorigenic capacity of PDAC was further determined using the colony formation assay and in vivo xenograft assays. Accordingly, TGIF1 ablation significantly enhanced the formation of tumor colonies in soft agar, suggesting the increased anchorage-independent growth ability of PDAC cells upon TGIF1 loss (Fig. 5e). Meanwhile, the tumor sphere formation assays also demonstrated that TGIF1 loss significantly increased the tumor sphere-forming ability of PDAC cells with or without TGFβ1 treatment (Fig. 5f, Additional file 2: Figure S2B). Furthermore, tumor SCID or allogeneic graft studies revealed that TGIF1 inactivation resulted in increased tumor incidence and size (Fig. 5Gi-ii, Additional file 2: Figure S3).

Next, to determine the influence of TGIF1 loss on the motility and invasiveness of PDAC cells, in vitro cell motility and invasiveness experiments were performed using wound closure and transwell migration assays. Our results revealed that the invasive ability of TGIF1-null PDAC cells was significantly higher than that of TGIF1-sufficient PDAC cells, as demonstrated by the wound healing assay results presented in Fig. 5h, and that TGIF1 loss markedly enhanced the migratory ability of PDAC cells. Consistent with this finding, the transwell migration assays revealed that TGIF1 deficiency led to an increase in the in vitro invasive ability of PDAC cells (Fig. 5i). Meanwhile, we obtained similar suppressive results of TGIF1 knockdown in the human Panc-1 PDAC cells (Additional file 2: Figure S4).

Similarly, the EMT has been described as an important process whereby tumor cells convert into prometastatic phenotypes. In the current study, we observed an appreciable induction of EMT upon TGIF1 ablation in PDAC cells. Through western blotting analysis, we further demonstrated the increased expression levels of p-Akt, p-STAT3, p-Erk (p44/42) an p-p38 MAP kinase pathways by the reduction of p-AMPK pathway in TGIF1-deficient PDAC cells as compared to TGIF1-sufficient PKP cells (Fig. 5J-i). Furthermore, to explore the functions of TGIF1 in regulating cancer stem cell (CSC) properties, we compared the expression levels of stemness transcriptional factors, such as Sox2, Nanog, CD44, Nestin, and CD133, between the TGIF1-deficient PDAC cells and TGIF1-sufficient PDAC cells. Our results indicated that TGIF1-null PDAC cells expressed higher levels of Nanog, Sox2, Nestin, CD44 and CD133 than TGIF1-sufficient PDAC cells (Fig. 5J-ii). Similar results were obtained by immunocytochemistry (Fig. 5k). Overall, our results indicate that TGIF1 loss enhances the EMT phenotype and plays an important role in the induction of CSC activity in PDAC.

### TGIF1 loss stimulates ETV1 transcription and activates Has2 expression in the KrasG12D p53 loxp/loxp PDAC model

**Fig. 6:**
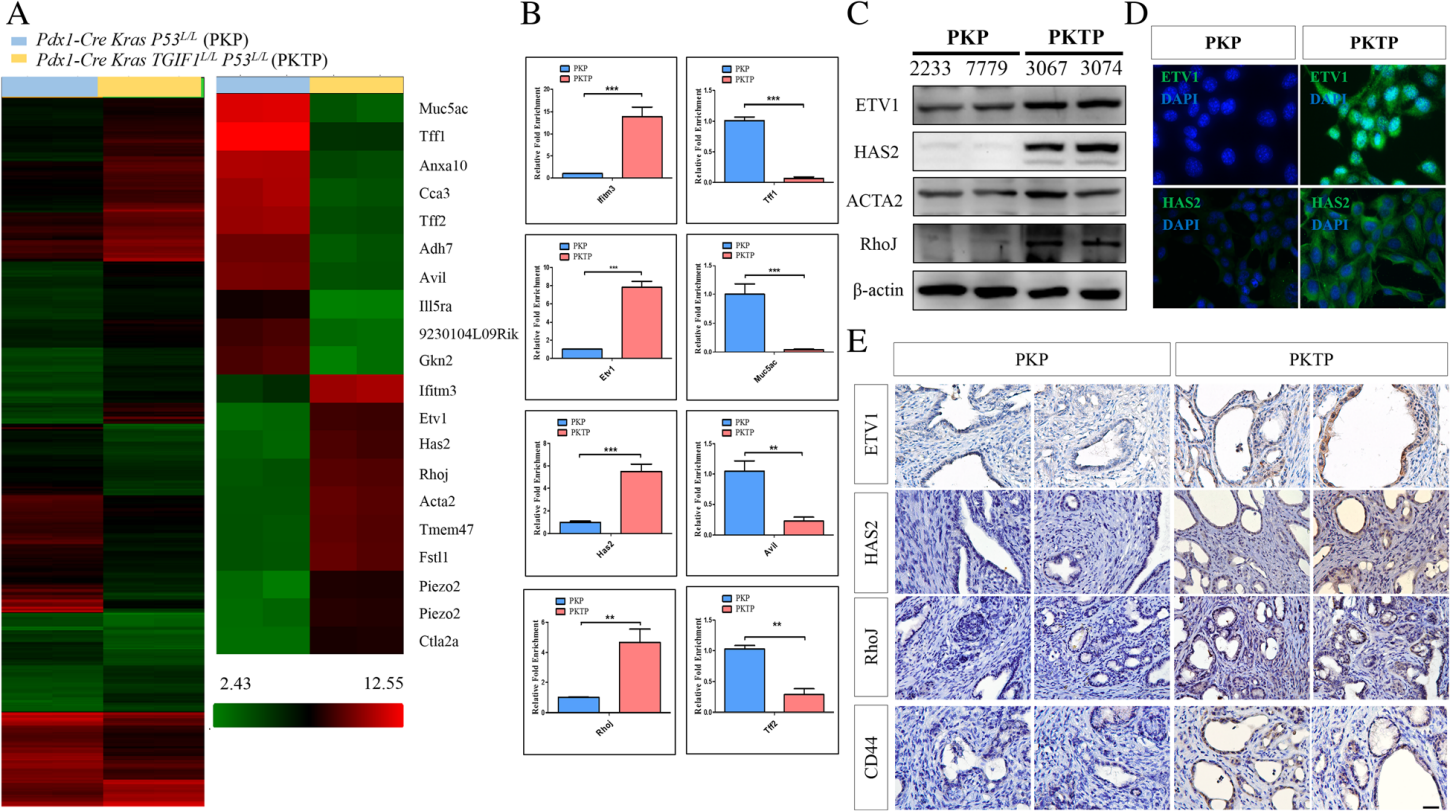
cDNA microarray analysis compared the differential gene expression of murine primary PDAC cells derived from PKP and PKTP mice. **a** Heat map showing the changes in gene expression in response to TGIF1 loss in PDAC. *P* < 0.05 was considered significantly differential gene expression. The scale bar extends the expression level of the gene from the florescence ratios of 2.43–12.55 Red, high expression level compared with the means. Green, low expression level compared with the means. Heatmap of the selected top 10 genes that were upregulated or downregulated in TGIF1-null PDAC cells as compared with TGIF1-sufficient PDAC cells. **b** RT qPCR analysis confirmed that the mRNA levels of IFITM3, ETV1, HAS2, and RHOJ were upregulated; whereas TFF1, MUC5AC, AVI1 andTFF2 were downregulated in PKTP cells, compared with PKP cells. The data were normalized to glyceraldehyde 3-phosphate dehydrogenase. Results are presented as mean ± SD of triplicates **p < 0.01; ***p < 0.001. **c** Western blot assays were performed to demonstrate the increased Etv1, Has2, RhoJ and Acta2 protein expression in PKTP cells as compared with the PKP cells. **d** Immunocytochemical detection of Etv1 and HAS2 expression in in PKTP cells as compared with the PKP cells. **e** IHC examination with antibodies against ETV1, HAS2, RhoJ and CD44 confirmed the increased expression of ETV1 and HAS2, RhoJ and CD44 in TGIF1-deficient PDAC as compared to TGIF1-proficient PDAC. Representative images are shown (× 500)

To investigate the mechanisms underlying TGIF1 loss-enhanced PDAC progression, we conducted a cDNA microarray analysis to compare the gene expression profiles between PKP and PKTP PDAC cells. Raw data and data of a detailed analysis on the intensities of the expressed gene were uploaded to the Gene Expression Omnibus (GEO) database (GEO accession number GSE108843). As shown in Fig. 6a (the top 10 regulated gene list), after TGIF1 loss, a total of 2069 differentially expressed genes (fold change > 2) were observed, comprising 1176 upregulated genes and 893 downregulated genes. The microarray assays indicated that the ETV1 (37.96-fold increase, PKTP vs PKP; *P* = 0.0004), HAS2 (37.33-fold increase, PKTP vs PKP; *P* = 0.001), IFITM3, PIEZO2, FSTL1, ACTA2, TMEM47, and RHOJ mRNA expression levels in TGIF1-deficient PDAC cells were upregulated. By contrast, the RNA levels of MUC5AC (240.62-fold decrease, PKTP vs PKP; *P* = 0.003), TFF1, GKN2, ANXA10, CLCA3, TFF2, and TGIF1 (29.09-fold decrease, PKTP vs PKP; *P* = 0.0007) were downregulated in TGIF1-deficient PDAC cells (GEO accession number GSE108843). The GenGo pathway analysis divided the upregulated genes into several categories according to the biological functions they are involved in: Cell cycle_the metaphase checkpoint, Cell adhesion_extracellular matric (ECM) remodeling, Cell cycle_DNA replication in the early S phase, and Development_Regulation of EMT. By contrast, the downregulated genes were involved in glutathione metabolism and metabolism regulation (bile acid regulation of glucose and lipid metabolism). We further confirmed the cDNA microarray results through RT-qPCR, Western blotting, and IHC analysis. RT-qPCR analyses verified the top regulated genes identified in the cDNA microarray study. RT-qPCR revealed a significant upregulation of IFITM3, ETV1, HAS2 and RHOJ mRNA in the TGIF1-deficient PDAC cells when compared with the PKP PDAC cells (Fig. 6b). Similarly, the Western blot analysis showed that the protein expression levels of Etv1, Has2, RhoJ and Acta2 were significantly increased in the TGIF1-deficient (PKTP) PDAC cells when compared with the TGIF1-sufficient PDAC (PKP) cells (Fig. 6c). These observations were also confirmed by immunocytochemistry and IHC detections in murine models (Fig. 6 d, e). The correlation between TGIF1 immunoreactivity and its downstream target genes, HAS2 and ETV1 expression patterns was further confirmed in human PDAC tissues by IHC analysis (Additional file 2: Figure S5). Collectively, the cDNA microarray data delineate that the upregulation of ETV1 and HAS2 signaling pathways might be responsible for the TGIF1 ablation-induced metastasis of PDAC.

### TGIF1 loss is associated with the epigenetic regulation of tumorigenesis in PDAC

**Fig. 7:**
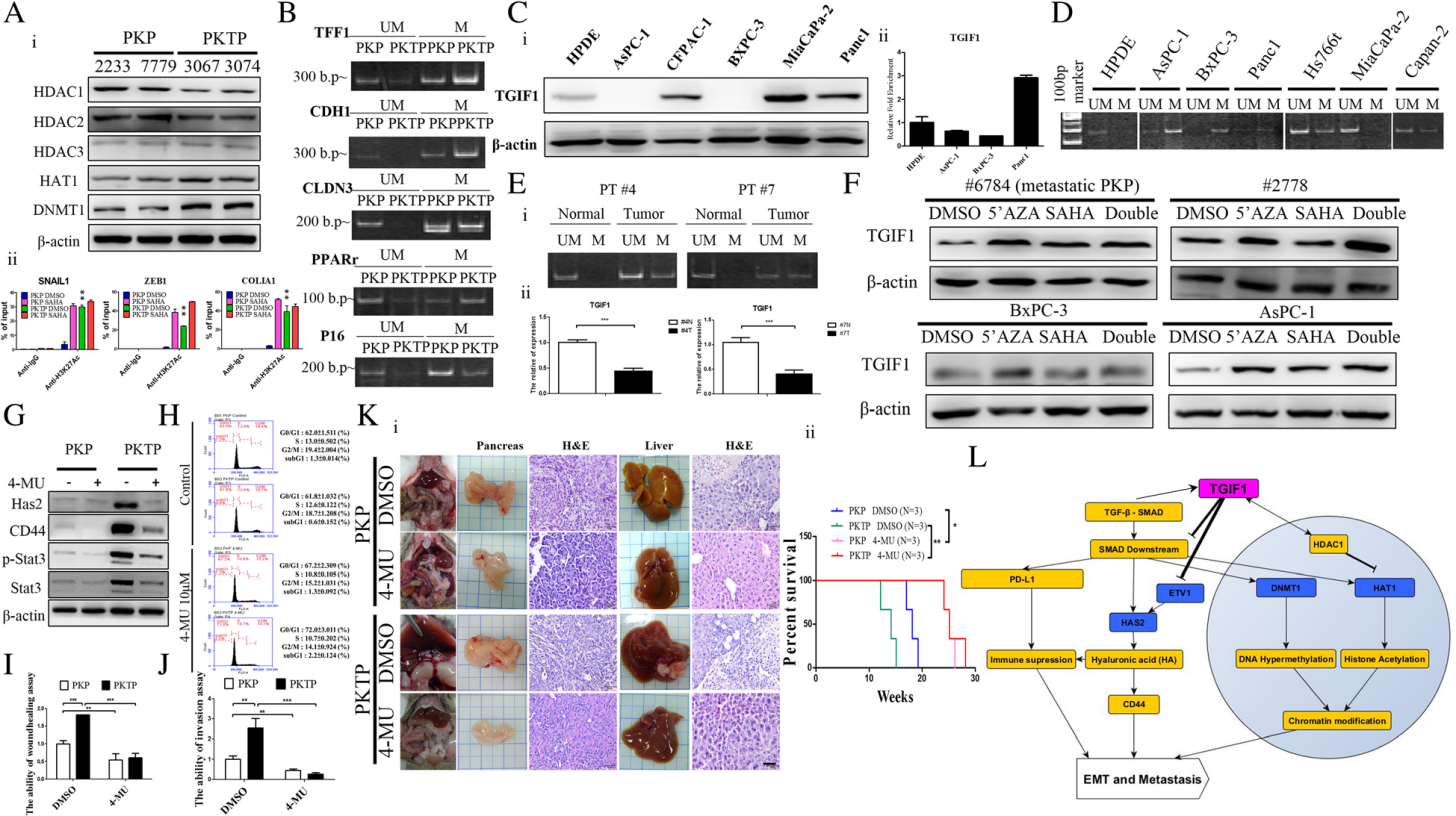
Epigenetic modulation of TGIF1 and targeting HAS2 suppresses TGIF1 loss-induced PDAC cell migration in vitro. **a i***,* Immunoblot analysis of HDAC1, HDAC2, HDAC3, DNMT1, and HAT1 from PKP and PKTP PDAC cells. **ii***,* Increase of histone H3K27 acetylation induced by TGIF1 loss binding on EMT associated SNAIL1, ZEB1 and COL1A1 promoter DNA of PKTP cells. Values are from three independent experiments with RT qPCR in duplicate. Bars, SD. IgG (2 μg/IP) was used as a negative IP control. The treatment with the commercial HDAC inhibitor SAHA (10 μM; 2 days) served as positive control for anti-acetyl-Histone H3K27 (Lys27) CHIP analysis. *P < 0.01. **b** Detection of TFF1, CDH1, CLDN3, PPARγand P16 promoter methylation statuses by using MSP in PKP and PKTP PDAC cells. **c** Determination of TGIF1 protein (**i**) and mRNA (**ii**) levels in different human PDAC cells through Western blot and RT-PCR analyses. **d** MSP analysis of the promoter region of TGIF1 in indicated human PDAC cell lines. UM, unmethylated primer pair; M, methylated primer pair. **e** The represented images of methylation status of the TGIF1 promoter in two pairs of human PDAC (tumor) and its adjacent normal pancreatic tissues (normal) by using MSP analysis (i), and their corresponding gene expression levels were determined by RT-qPCR(ii). The housekeeping gene glyceraldehyde 3-phosphate dehydrogenase (GAPDH) served as an internal control. **f** Effects of DNA methylation and histone acetylation on the regulation of TGIF1 gene expression. Effects of 5-Aza and SAHA on TGIF1 expression in PDAC cells. Restoration of TGIF1 expression in mouse 6784, 2778, human BxPC-3 and AsPC-1 cells treated with 5-Aza, SAHA alone or both were analyzed using western blot analysis, and β-actin used as loading controls. **g** western blot analysis of CD44, total and phosphorylated Stat3 in PKTP PDAC cells with or without 4-Methylumbelliferone (4-MU) treatment. **h** Representative flow cytometric analysis of cell cycle showing PKP and PKTP PDAC cells following 4-MU (10 μM) overnight treatment compared with methanol control groups. **i** Quantitative assessment of wound closure rates of PKTP PDAC cells were analyzed using wound healing assays with the treatment of 5 μM of the Has2 inhibitor, 4-MU. **j** Quantitative determination of transwell migration assays of PKP or PKTP PDAC cells treated or untreated with 4-MU (5 μM) for 18 h **p < 0.01; ***p < 0.001. **k** Effect of 4-MU on PDAC formation in vivo. i. Representative anatomical images of the abdomen of PKP and PKTP mice treated intraperitoneally with 4-MU or vehicle (DMSO) for 4 weeks showing the reduction of PDAC formation in the 4-MU treated PKP and PKTP mice, but not in the mice receiving DMSO treatment. Macroscopic appearance and H&E histological analysis of murine pancreas and liver after 4-MU treatment or DMSO control groups are shown. Scale bar, 50 μm*.* **ii**, Kaplan-Meier survival curves of PKP or PKTP mice with or without 4-MU. PDAC mice treated with 4-MU significantly live longer than untreated mice. *P < 0.05; **P < 0.01 **l** The schematic diagram showing the roles of TGIF1 in the TGFβ1/Smad signaling in modulating EMT program and tumor metastasis

TGIF1 has been demonstrated to interact with HDAC, and TGIF1 knockdown reduces HDAC1 protein expression [20]. In the present study, we found that TGIF1 loss increased HAT1, DNMT1 expression but reduced HDAC1 protein expression in our model (Fig. 7a). Considering this finding, we hypothesized that TGIF1 loss is associated with epigenetic modifications in PDAC. Experiments were first conducted, confirming that TGIF loss in PDAC cells reduced the HDAC1/2 levels but increased HAT1, DNMT1 expression, as revealed by western blotting (Fig. 7Ai). In addition, TGIF1 loss induces high levels of H3K27 acetylation (H3K27ac) in the SNAIL1, ZEB1 and alpha-1 type I collagen (COL1A1) promoter DNA of murine PDAC cells (Fig. 7Aii). Methylation-specific PCR (MSP) analysis revealed the increased promoter hypermethylation of TFF1, E-cadherin (CDH1), CLDN3, and PPARγ genes, which were shown to be downregulated in TGIF1-null PKTP cells when compared to PKP cells in our cDNA microarray analysis (Fig. 7b). These data imply that TGIF1 may play a role in regulating chromosome DNA methylation and histone acetylation in PDAC.

Furthermore, we tend to determine the molecular mechanisms underlying TGIF1 inactivation and loss of expression during PDAC development. We initially considered that epigenetic regulation might serve as a key mechanism for TGIF1 silencing. To explore whether the epigenetic mechanism leads to the loss of TGIF1 expression in PDAC, we postulated that the TGIF1 upstream promoter sequences CpG islands may be the targets for DNA hypermethylation to silence TGIF1 gene expression in PDAC. To determine whether DNA hypermethylation alone or combined with chromatin acetylation can affect TGIF1 gene expression, we selected murine and human BxPC-3 and AsPC-1 PDAC cell lines, which exhibited low TGIF1 protein expression, to investigate the methylation-mediated TGIF1 gene expression (Fig. 7c). As shown in Fig. 7 d and e, the MSP analysis of the target CpG islands in the promoter region of the TGIF1 gene demonstrated that the promoter DNA was hypermethylated in the PDAC cell lines and primary human PDAC tissues with a low or negative expression of TGIF1. Next, we further treated the cells with the DNA demethylated agent 5-aza-2′-deoxycytidine (5′AZA) alone or 5′AZA combined with suberoylanilide hydroxamic acid (SAHA), a DNA deactylation agent, and performed RT-PCR to demonstrate TGIF1 reactivation (Fig. 7f). Our data suggest that the loss of TGIF1 expression in cancers is primarily mediated by the hypermethylation of promoter CpG islands of the gene in PDAC.

### Assessment effect of the Has2 inhibitor 4-methylumbelliferone (4-MU) on HA/CD44 signaling, growth, and migration of PDAC cells in vitro

Similar to our finding in this study, a recent study demonstrated that Etv1 enhances Has2 expression in pancreatic cancer and alters the tumor microenvironment [31]. Moreover, the Has2 enzyme is involved in hyaluronan (HA) synthesis and enhances the HA/CD44 cancer stemness pathway. This prompted us to determine whether Has2 inhibition could affect TGIF1 loss-induced HAS/CD44 signaling in PDAC. For this purpose, we first demonstrated that stable knockdown of ETV1 or HAS2 inhibits CD44 expression and reduces in vitro cell migration in PKTP cells (Additional file 2: Figure S6), and afterwards we treated primary murine PDAC cell lines with the Has2 inhibitor 4-methylumbelliferone (4-MU), and Has2, p-STAT3, and CD44 protein levels were determined using immunoblot analyses to confirm the drug effectiveness [32]. As shown in Fig. 7g, 4-MU reduced the protein levels of Has2, p-Stat3 and CD44. Next, we investigated the effect of 4-MU on cell cycle progression by using flow cytometry analysis; as shown in Fig. 7h, the inhibition of Has2 activity significantly reduced the percentage of S phase cells and increased the percentage of G0/G1 phase cells in PKTP and PKP PDAC cells (Fig. 7h).

Subsequently, we assessed the effect of Has2 inhibitors on the suppression of cell motility in vitro through wound closure and transwell invasion analysis. The inhibition of Has2 activity by 4-MU significantly reduced the migration rates of the PKTP cells in the cell scratch assay (Fig. 7i). We also observed similar results when the invasive ability of the PKTP cells was appreciably inhibited by 4-MU in the in vitro transwell assays (Fig. 7j). Lastly, we further showed that in vivo treatment with 4-MU significantly inhibited PDAC formation and metastasis in PKP^(P53Loxp/+)^ and PKTP^(P53Loxp/+)^ models (Fig. 7Ki-ii). Collectively, these findings suggest that a novel Has2/CD44 metastatic pathway mediated by TGIF1 loss is involved in promoting PDAC metastasis.

## Discussion

PDAC is highly invasive, with prevalent peritoneal metastasis, that is commonly characterized by metastasis to the liver and more distant organs [33]. Notably, human PDAC often characteristically presents with a substantial stromal component, and in some late-stage lesions, the transformed epithelium represents a small fraction of the tumoral mass [34]. Among various cytokines associated with PDAC development, TGFβ ligands and their receptors, induced in low-grade PanINs, are perceptible markers of PC progression, suggesting that the signaling of the TGFβ family may contribute to the earliest stages of pancreatic ductal neoplasia [35]. The TGFβ1 pathway is likely to contribute to the maintenance of established tumors, because disruption of TGFβ1/SMAD signaling in human PDAC cells influences cancer cell growth in vitro and tumorigenesis in xenografts [36]. In contrast, the enhanced oncogenic effects of TGFβ1 signaling in PDAC are likely to be directed by numerous genetic or epigenetic events, including TGIF1 inactivation that might alter tumor cell survival and proliferative as well as metastatic processes, because TGIF1 can cause feedback regulation of TGF-β signaling to influence tumor cell proliferation and promote malignant transformation of the tumor microenvironment. [21].

In this paper, we first report that TGIF1 is appreciably inactivated during PDAC progression and suggest that TGIF1 might serve as a potential prognostic indicator for PDAC progression. To explore the effects of TGIF1 inactivation on pancreatic development and tumorigenesis, we incorporated a conditional TGIF1 deletion strain into the Pdx-1Cre mouse model. Consequently, we observed that conditional TGIF1 deletion under Pdx-1Cre-driven induction appears to develop normally with normal pancreatic functions in mice. Previously, we and other researchers have developed a mouse model of PDAC, a histological subtype of epithelial PDAC, which mimics the human histological phenotype. Our studies, particularly the present study, unequivocally demonstrated the cooperative effects of Kras^G12D^ activation and TGIF1 deletion in premalignant pancreatic ducts and neoplastic derivatives. We observed that Pdx-1CreKras^G12D^ mice harboring this latent allele developed PanIN lesions by the age of > 12 months, which is consistent with the premalignant transformation of the pancreatic ductal epithelium. When crossed with a strain harboring an LSL-Kras^G12D^ allele combined with the conditional TGIF1 alleles rendered null in the pancreas by Pdx-1-directed Cre recombinase activity, these PKT mutant mice exhibited malignant progression of the PanINs rapidly to PDAC, which have a median survival time of < 18 weeks. Additionally, our analysis of PKTP mice indicated the presence of widely metastatic PDAC as early as 6 weeks of age, with the mice having a median survival time of < 6 weeks; this confirms that TGIF1 inactivation results in rapid PDAC progression in mice. In this context, we observed that more than 60% of our PKTP mice had distant lung metastasis, whereas only 17% of the PKP mice did, which demonstrates that TGIF1 inactivation in TGFβ1/Smad signaling might both increase malignancy and enhance distant metastasis. Interestingly, patients whose pancreatic cancer had elevated TβR2 expression exhibited reduced overall survival compared with patients whose pancreatic cancers had lower levels of TβR2 from the Cancer Genome Atlas (TCGA public data portal: http://tcga-data.nci.nih.gov/). Meanwhile, TGFβ has been shown to promote PDAC desmoplasia as well as contribute to proliferation and invasion of tumor cell subsets in an autocrine manner; notably blockade of TGFβ signaling attenuates tumorigenicity of some xenografts. The TGIF1 tumor suppressor protein functions as a critical repressor of the TGF-β1-SMAD signaling pathway. TGF-β1-dependent transcriptional repression by TGIF1 is mediated by competition with the co-activator p300/CBP for Smad2 interaction, thereby repressing TGF-β signal transduction and downstream gene expression [20]. In addition, and independently of Smad signaling, TGIF1 has been reported to be a necessary component of the tumor necrosis factor α (TNF-α) cytotoxic program [37]. Thus, the malignant and metastatic phenotype of TGIF1 deficient PDAC could be distinguished from those with TβR2 mutant (reported by Ijichi et al. *Genes Dev* 2006) by virtue of their pleiotropic tumor promoting effects mediated by different pro-inflammatory cytokines [38], and according to our review of the relevant literatures, this is the first in vivo study to report that TGIF1 ablation in the TGFβ1-Smad pathway promotes the development of PDAC.

Tumor metastasis involves the degradation of an existing ECM, morphological and cytoskeletal changes at the tissue and cell levels, and activation of a motility response to promote cell migration [39, 40]. Our microarray analysis conducted on primary PKTP and PKP PDAC cells demonstrated the top gene candidates regulated by TGIF1 involved in cancer stemness or the prometastatic category might be associated with their roles in mediating EMT and distant metastasis of PDAC. Certainly, Evt1, Has2, Acta2, Rhoj, Thbs1, and Tnc are associated with EMT transition, and virtually most of our top candidates appear to be important for ECM and actin remodeling. Of these genes, RHOJ and FSTL1 have particularly been characterized more extensively, whereas ETV1 and HAS2 are newly emerging in the cancer literature [41, 42]. In particular, the ETS family transcription factor ETV1, a member of the polyoma enhancer activator 3 (PEA3) Ets transcription factors, has been shown to govern EMT, activate the stromal population, and promote tumor metastasis [43, 44]. A study of the clinical effect of Etv1 demonstrated that the ETV1-positive group was associated with a markedly poor overall survival in triple-negative breast cancer [42]. Li and colleagues reported that ETV1 promotes Snail expression to induce EMT-like metastatic progression in gastric cancer [44]. In addition, research has proposed that HAS2 affects hyaluronic acid synthesis as a result of HA/CD44 signaling pathway activation to influence tumor microenvironment transformation [45, 46]. In the present study, we propose that Etv1 is a nuclear transcriptional factor suppressed by the TGIF1 repressor, and Etv1 upregulation mediated by TGIF1 ablation confers to increase Has2 activity and activate the HA/CD44 cancer stemness pathway in PDAC. Numerous reports have indicated that CD44 is related to cancer stemness activity [47]. CD44, which can selectively bind to HA, has been identified by several studies to play crucial roles in cancer proliferation, invasion, and metastasis in various cancers [46, 48]. For instance, CD44 is highly expressed in metastatic breast cancer and is chemoresistant to doxorubicin, whereas it is expressed in low amounts or is absent in normal breast tissue [49, 50]. In addition, increased CD44 expression in colorectal cancer is associated with the aggressive behavior of colorectal cancer, indicating that it may play potential roles in cancer stemness traits [51]. Furthermore, HA production has also been implicated in the regulation of inflammatory stimuli and possible suppression of immunogenicity through potential interfering effects of HA and immune cells or other antitumor glycosaminoglycans; for example, high HA binding on activated T cells can trigger cell death [52].

In recent years, the immune response in cancer pathology has become a prevalent target for therapeutics. Several studies have elucidated the molecular associations between immune checkpoint blockade and tumor development, and recently, the influence of TAMs on the tumor microenvironment has received increased attention because they play pivotal roles in the modulation of tumor immunogenicity, angiogenesis, chemoresistance and metastasis [53]. To put this into perspective, high HAS2 expression levels in breast CSCs have been shown to confer TAMs M1/M2 polarization in the tumor microenvironment of breast cancer and promote breast cancer with bone metastasis [46]. In particular, polarization of macrophages to the M2-like activation state increases the production of IL-4 and M-CSF as well as upregulation of several chemokines including CCL1, CCL24, and CCL17; furthermore, M2 macrophages play a crucial role in mediating tissue remodeling, immune regulation, and tumor promotion [54]. In the current study, we observed that TGIF1 inactivation promoted the production of IL-4, M-CSF, CCL1, and CCL24 in murine PDAC, which has been implicated in the conversion of TAMs to M2-like phenotypes. Considering these findings, we propose that the upregulation of HAS2 induced by TGIF1 loss in PDAC may involve in the stimulation of TAM polarization to M2-like alternative activated macrophages, thus enhancing the development of PDAC in our models. Moreover, we identified a novel interaction of TGIF1 with the immune inhibitory molecule PD-L1 and demonstrated that TGIF1 ablation is associated with PD-L1 upregulation in PDAC. PD-L1 regulates the immune checkpoint and controls T-cell-mediated host antitumor immune responses. Additionally, PD-L1 overexpression in tumor tissues may implicate a negative prognostic factor and a potential maker for metastasis in patients with cancer [55, 56].

We further detected that TGIF1 DNA promoter hypermethylation in a certain fraction of primary human PDAC in close association with TGIF1 expression loss or TGIF1 downregulation. According to this finding, we hypothesize that the epigenetic silencing of TGIF1 expression is a frequent event in the development of PDAC. Additional studies are warranted to determine its expression status and methylation status in association with its expression in other types of cancer. Moreover, recent studies have suggested that TGFβ1 signaling may be involved in the epigenetic regulation of EMT and DNA methylation maintenance during tumor progression [57, 58]. Similarly, we observed that TGIF1 loss leads to an increase in the DNA hypermethylation of genes involved in epithelial cell polarity, cell adhesion, or tight junction formation, such as E-cad and TFF1. These observations are consistent with those of a recent study that demonstrated that the TGFβ1-Smad signaling axis is responsible for the maintenance of specific epigenetic modifications that favor the EMT and prometastatic phenotypes in breast cancer. Thus, TGIF1 may play a pivotal role in TGFβ1-mediated epigenetic programming to regulate DNA methylation involved in the regulation of gene transcription during PDAC development.

In summary, in the current study, we used PDAC-prone mouse models combined with histopathological and genome-wide gene profile analyses to explore the spectrum of alterations in gene expression by the genetic ablation of TGIF1 in murine PDAC models. We demonstrated that TGIF1 inactivation promotes Kras^G12D^-driven pancreatic tumorigenesis and that TGIF1 ablation has tumor-promoting effects and activates the HAS2/CD44 cancer stemness pathway, a potent regulator in the induction of TAM polarization, which enhances PDAC distant metastasis. Moreover, our results reveal a novel role of TGIF1 in suppressing the expression of PD-L1, an immune checkpoint molecule, and modulating epigenetic changes in PDAC. Taken together, we highlighted key effectors of TGIF1 loss in murine PDAC biology and determined that TGIF1 loss led to more aggressive, EMT-high, and elevated stemness gene signatures in PDAC (Fig. 7l). Our data also provide preclinical evidence supporting the new strategy of identifying new small inhibitors to block the HAS2/CD44 pathway in order to eliminate distant metastasis in patients with PDAC harboring TGIF1 deficiency. Meanwhile, since PD-L1 protein is expressed at high levels in tumors of the PKTP mice (Fig. 4b), suggesting that administration of antibodies against PD-L1 or PD-1 into PKTP mice may attenuate PDAC growth.

## Conclusions

Our study here comprehensively characterized that TGIF1 functions as a PDAC tumor suppressor, blocking the progression of KrasG12D-initiated neoplasms, inhibiting EMT, modifying cancer epigenetics and tumor immune microenvironment, as well as restricting the activation of Has2/CD44 signaling pathway in facilitating distant metastasis. Our results suggest that Has2/CD44 signaling might be a valid target for patients with TGIF1-deficient pancreatic cancer.

## Additional files

Additional file 1: **Table S1.** List of the primary antibodies used in this study, and information on working dilutions of antibodies in Western blotting (WB), immunohistochemistry (IHC) and immunofluorescence (IF). **Table S2.**. List of real-time PCR primers used in the study. **Table S3**. List of the MSP primers used in this study. **Table S4**. Comparison of incidence of metastatic lesions present in various organs of PKP and PKTP mouse models. (XLSX 21 kb)

Additional file 2: **Figure S1.** TGIF1 downregulation is associated with tumor progression in human PDAC. **Figure S2.** Knockdown of TGIF1 displays resistant to TGFβ1 mediated growth inhibition in PDAC. **Figure S3.** TGIF1 loss exhibits increased in vivo tumorigenic potential of PDAC cells in an allogeneic tumor graft model. **Figure S4.** Knockdown of TGIF1 enhances tumor sphere forming and migratory abilities in human Panc-1 PDAC cells. **Figure S5.** Inactivation of TGIF1 upregulated HAS2 and ETV1 expression in human pancreatic tissues as determined by IHC analysis. **Figure S6.** Knockdown of ETV1 or HAS2 inhibits CD44 expression and reduces in vitro cell migration in PKTP 3067 cells. (DOCX 18253 kb)

## Abbreviations

4-MU: 4-Methylumbelliferone
CHIP: Chromatin immunoprecipitation
EMT: Epithelial–mesenchymal transition
GEM: Genetically engineered mouse
GTT: Glucose tolerance test
HAS2: Hyaluronan synthase 2
IF: Immunofluorescence
IHC: Immunohistochemistry
MSP: Methylation-specific polymerase chain reaction
PC: Pancreatic cancer
PDAC: Pancreatic ductal adenocarcinoma
PD-L1: Programmed cell death receptor ligand 1
PK: Pdx-1CreKras^G12D^
PKP: Pdx-1CreLSL-Kras^G12D^p53^L/L^
PKT: Pdx-1CreKras^G12D^TGIF1^L/L^
PKTP: Pdx-1CreKras^G12D^ TGIF1^L/L^p53^L/L^
SAHA: Suberoylanilide hydroxamic acid
TGIF1: TG-interacting factor 1
