# Supplementary material for: Loss of the transcriptional repressor TGIF1 results in enhanced Kras-driven development of pancreatic cancer
Source: Mol Cancer. 2019 May 20;18:96. doi: 10.1186/s12943-019-1023-1 (PMC6526617; doi:10.1186/s12943-019-1023-1)
Supplement: Supplementary file 2 — Figure S1. TGIF1 downregulation is associated with tumor progression in human PDAC. Figure S2. Knockdown of TGIF1 displays resistant to TGFβ1 mediated growth inhibition in PDAC. Figure S3. TGIF1 loss exhibits increased in vivo tumorigenic potential of PDAC cells in an allogeneic tumor graft model. Figure S4. Knockdown of TGIF1 enhances tumor sphere forming and migratory abilities in human Panc-1 PDAC cells. Figure S5. Inactivation of TGIF1 upregulated HAS2 and ETV1 expression in human pancreatic tissues as determined by IHC analysis. Figure S6. Knockdown of ETV1 or HAS2 inhibits CD44 expression and reduces in vitro cell migration in PKTP 3067 cells. (DOCX 18253 kb) [file 12943_2019_1023_MOESM2_ESM.docx]

**Supplementary figure legends**


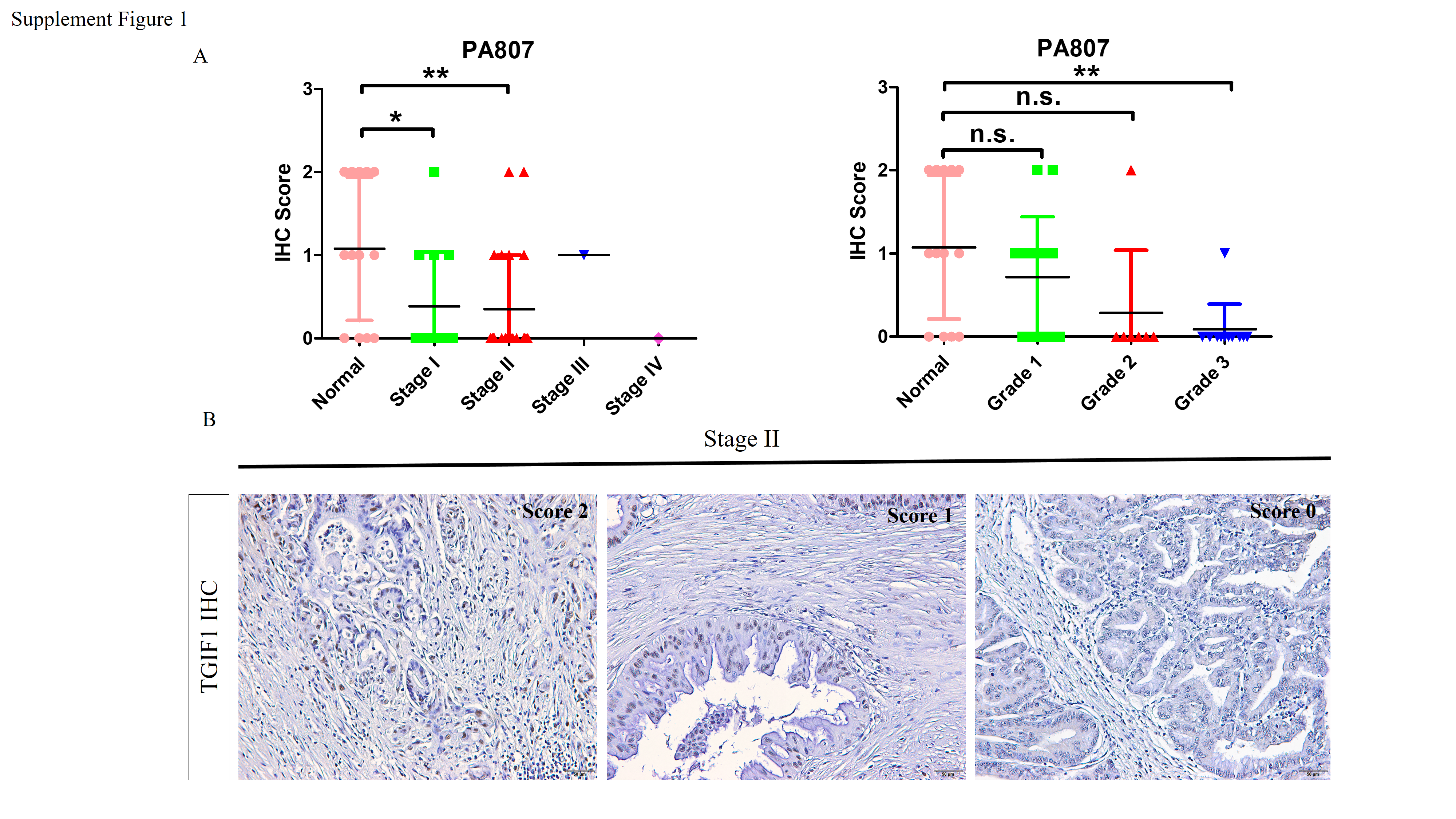


**Figure S1. TGIF1 downregulation is associated with tumor progression in human PDAC. Supplementary Figure S1. TGIF1 downregulation is associated with tumor progression in human PDAC.** A**,** Quantitation of IHC analysis for human TGIF1 expression in tissue microarray (TMA) analysis, and the correlation between patient clinical features and TGIF1 expression normal versus tumor ratio in a pancreatic carcinoma tissue microarray (TMA). A Mann–Whitney test was also used to compare normal with stage I (P = 0.05), normal with stage II (P = 0.01). The samples between stage III and stage IV (N=2) is not significant in this study. B**,** Human PDAC TMA spots stained for TGIF1 were interpreted by pathologist visual scoring as 0 (no staining), 1 (<20% of PDAC staining), 2 (>20% of PDAC staining). Representative spot in stage II for each score is shown.


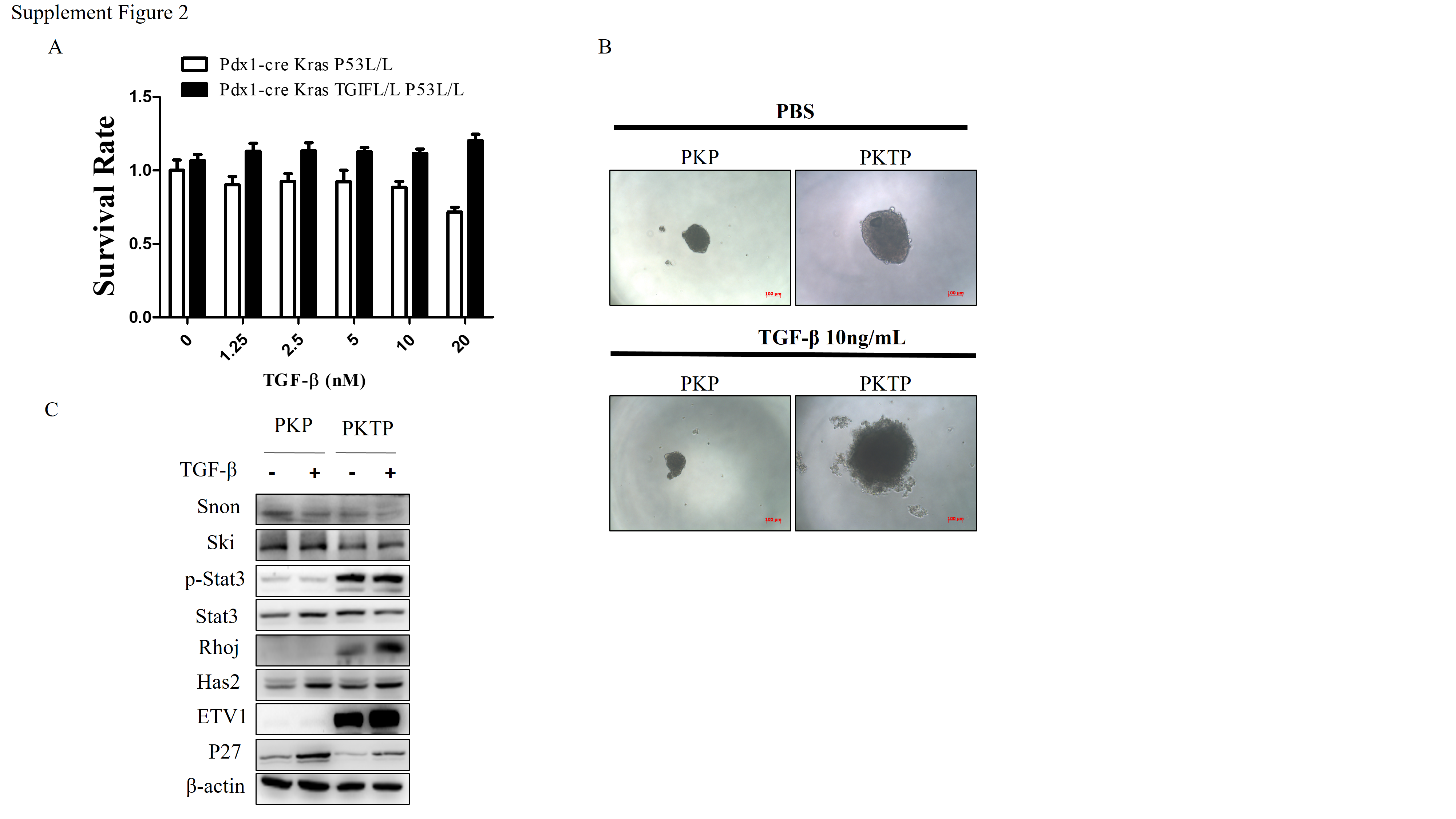


**Figure S2. Knockdown of TGIF1 displays resistant t**o **TGFβ1 mediated growth inhibition in PDAC.** A, TGIF1 knockdown blocks TGF-β1 induced cell cycle arrest in murine PDAC cells. The cell survival analysis demonstrated the growth inhibition of TGFβ1 treated PKP cells, but not PKTP cells treated with TGFβ1. PKTP cells are resistant to TGFβ1 mediated growth inhibition as compared to PKP cells. B, TGFβ1 promotes tumor spheroid growth of PKTP cells in hanging drop experiments, but not in PKP cells. C, Western blotting revealed that altered expression levels of TGFβ1 repressors SnoN and Ski, Has2, Etv1, RhoJ, p-STAT3 and total STAT3 protein expression when compared with PKP and PKTP PDAC cells with or without TGFβ1 treatment. P27 was a potent TGFβ1 regulator to confirm the TGFβ1 treatment. β-actin was used as a loading control.


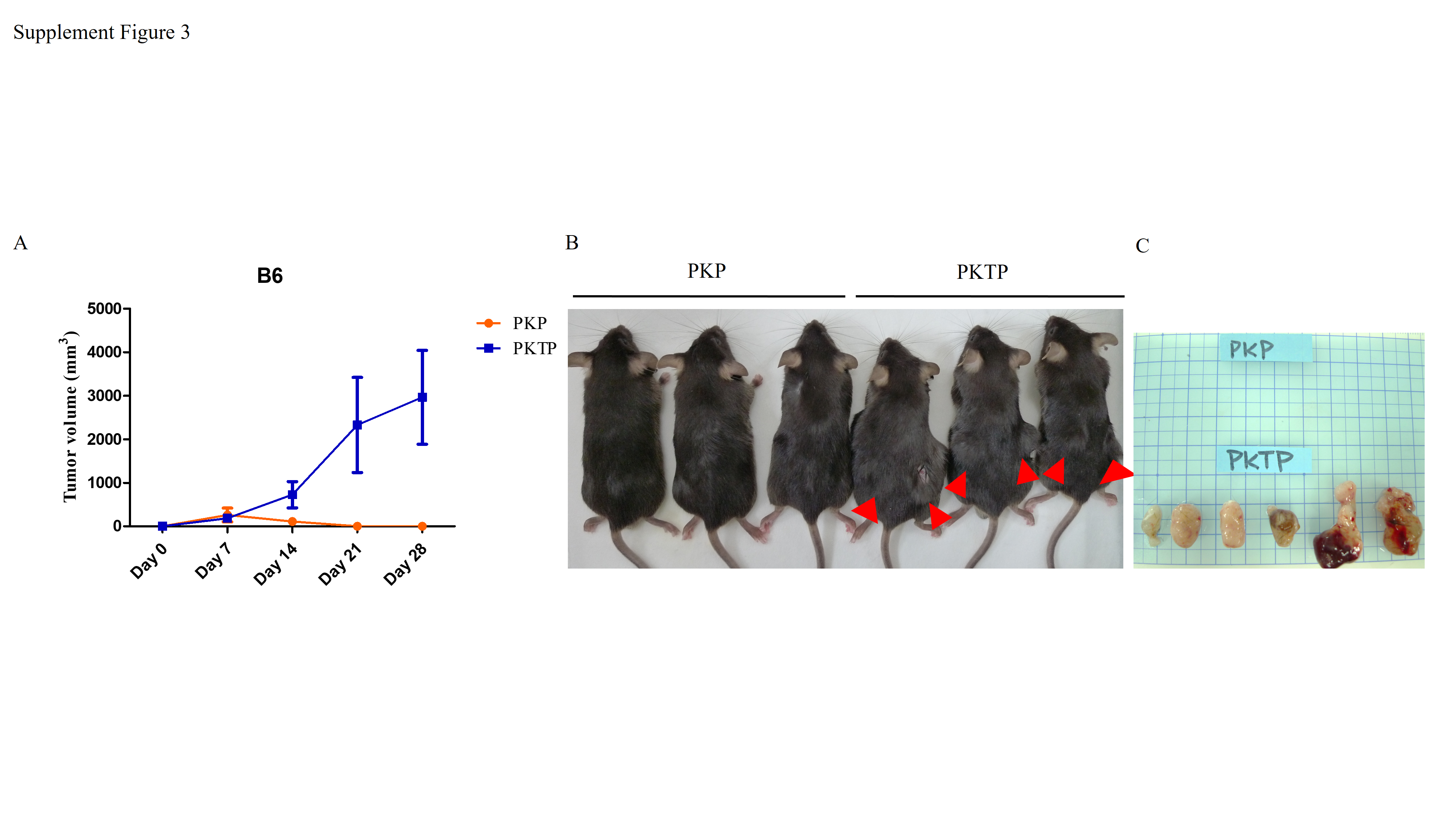


**Figure S3. TGIF1 loss exhibits increased in vivo tumorigenic potential of PDAC cells in an allogeneic tumor graft model.** A, Tumor growth curve after subcutaneous implantation of PKP and PKTP cells (FVB/129sv background) in C57BL6/J mice (N=6 each group). The tumors were measured for up to 30 days, and xenograft tumor volumes from indicated groups are presented. B&C, The allogeneic models (B) and xenograft tumor gross images (C) exhibit representative samples of PKP and PKTP tumors excised after sacrificing the mice on day 30 for both models. The results demonstrated that PKTP cells rapid form large tumors by subcutaneous (s.c) injection into murine allo-tumor graft models, whereas, PKP cells failed to form s.c tumors within 30 days in allogeneic graft model.


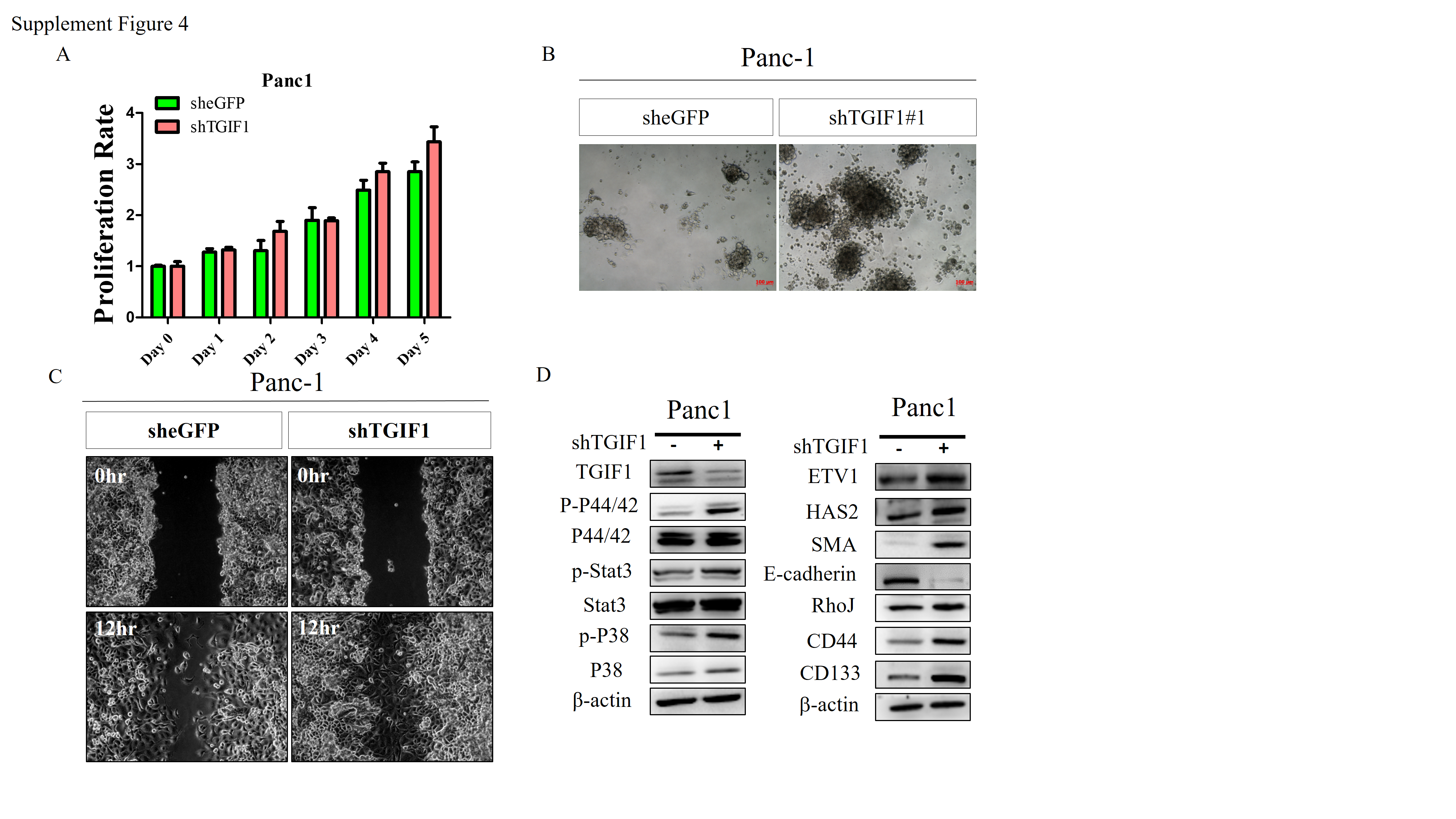


**Figure S4. Knockdown of TGIF1 enhances tumor sphere forming and migratory abilities in human Panc-1 PDAC cells.** A, Knockdown of TGIF1 does not affect the growth rate of Panc-1 cells as determined by in vitro cell proliferation assays. B, TGIF1 knockdown increases tumor sphere forming in hanging drop spheroid culture assays. C, Wound healing assays showed that knockdown of TGIF1 promoted cell motility in Panc-1 cells. Magnification, ×40. Representative results of three different experiments. D, Loss of TGIF1 increases Has2, Etv1, RhoJ, CD44, CD133, phosphorylated p44/42, p38 and STAT3 protein expression, and promotes EMT program in Panc-1 cells. β-actin was used as a loading control.


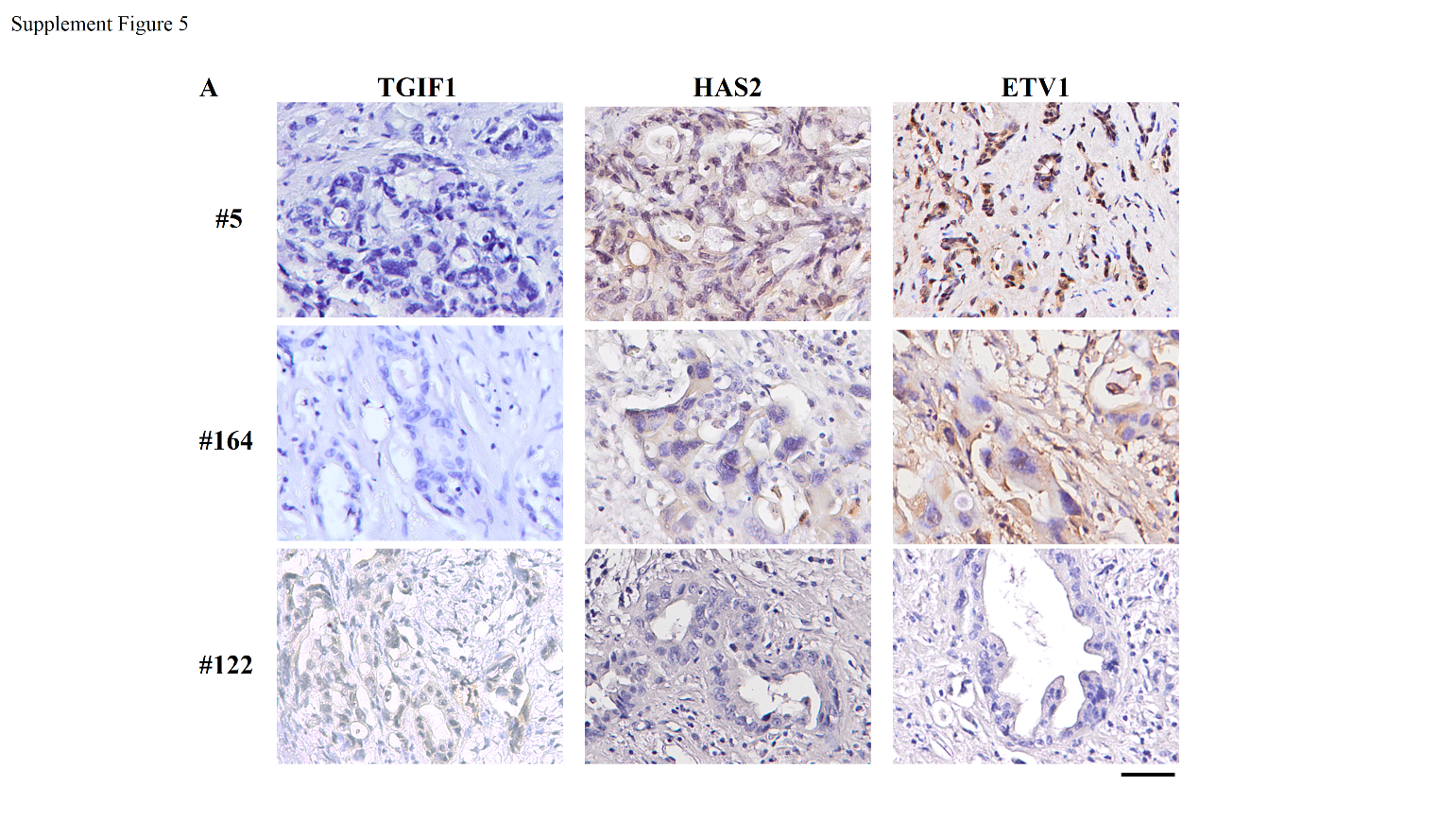


**Figure S5. Inactivation of TGIF1 upregulated HAS2 and ETV1 expression in human pancreatic tissues as determined by IHC analysis**. IHC analysis of the impact of TGIF1 expression on HAS2 and ETV1expression in human pancreatic tissues. In TGIF1-negative (patient #5) and (#164) and TGIF1-positive (#122) human PDAC tissues, serial sections were stained with anti-TGIF1, anti-HAS2 and anti-ETV1 antibodies for immunohistochemical analysis. Magnification, ×200; red bar, 50 μm.


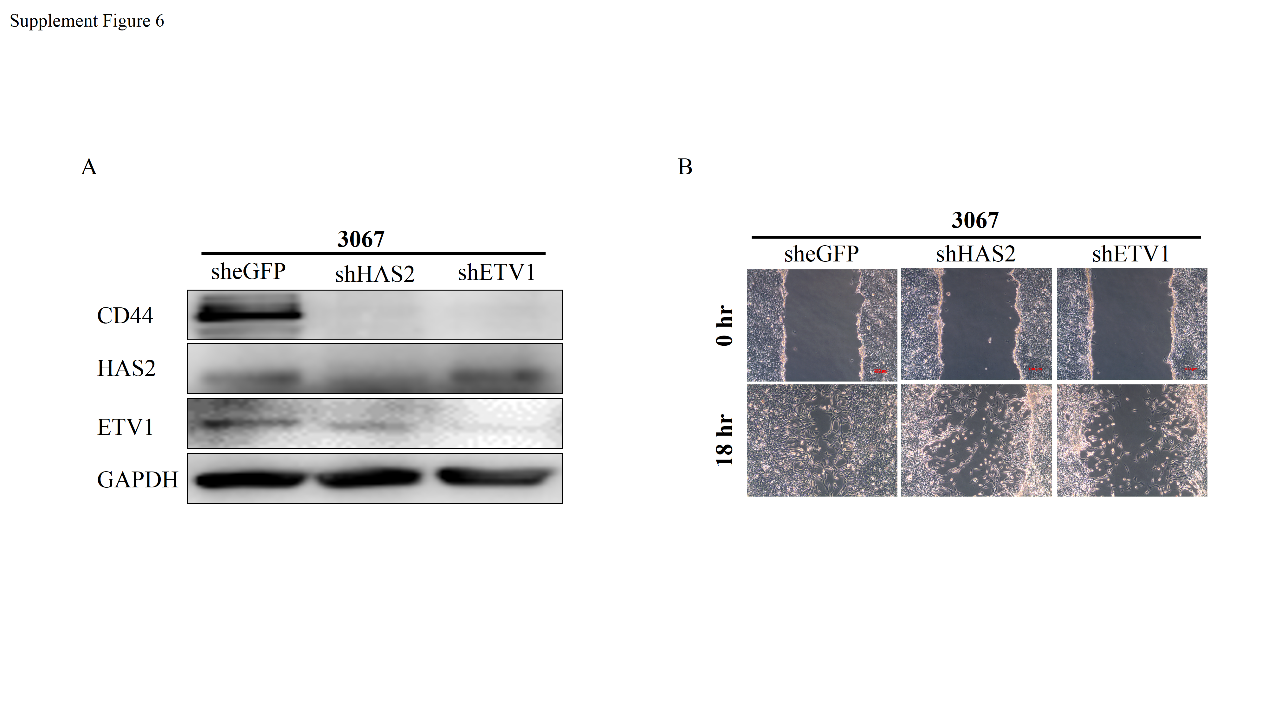


**Figure S6. Knockdown of ETV1 or HAS2 inhibits CD44 expression and reduces in vitro cell migration in PKTP 3067 cells.** A. western blot analysis of CD44, ETV1 and HAS2 protein expression in 3067 control sheGFP and shHAS2(clone TRCN0000337811) and shETV1 (TRCN0000075475) stable knockdown cells. B. knockdown of HAS2 or ETV1 reduces wound closure ability in 3067 PKTP cells as determined by in vitro wound scratch assays.
